# Extra-domain B of fibronectin as an alternative target for drug delivery and a cancer diagnostic and prognostic biomarker for malignant glioma

**DOI:** 10.7150/thno.44948

**Published:** 2021-01-01

**Authors:** Phei Er Saw, Xiaoding Xu, Bo Ram Kang, Jungsul Lee, Yeo Song Lee, Chungyeul Kim, Hyungsin Kim, Shin-Hyuk Kang, Yoo Jin Na, Hong Joo Moon, Joo Han Kim, Youn-Kwan Park, Wonki Yoon, Jong Hyun Kim, Taek-Hyun Kwon, Chulhee Choi, Sangyong Jon, Kyuha Chong

**Affiliations:** 1 Guangdong Provincial Key Laboratory of Malignant Tumor Epigenetics and Gene Regulation, Sun Yat-Sen Memorial Hospital, Sun Yat-Sen University, Guangzhou 510120, People's Republic of China; 2 Department of Biological Sciences, KAIST, 291 Daehak-ro, Yuseong-gu, Daejeon, 34141, Republic of Korea; 3 Photo-Theranosis and Bioinformatics for Tumor Laboratory, Korea University Guro Hospital, Korea University Medicine, Korea University College of Medicine, 148 Gurodong-ro, Guro-gu, Seoul, 08308, Republic of Korea; 4 Department of Neurosurgery, Korea University Guro Hospital, Korea University Medicine, Korea University College of Medicine, 148 Gurodong-ro, Guro-gu, Seoul, 08308, Republic of Korea; 5 Department of Bio and Brain Engineering, KAIST, 291 Daehak-ro, Yuseong-gu, Daejeon, 34141, Republic of Korea; 6 Department of Pathology, Korea University Guro Hospital, Korea University Medicine, Korea University College of Medicine, 148 Gurodong-ro, Guro-gu, Seoul, 08308, Republic of Korea; 7 Department of Neurosurgery, Korea University Anam Hospital, Korea University Medicine, Korea University College of Medicine, 73 Goryeodae-ro, Seoungbuk-gu, Seoul, 02841, Republic of Korea; 8 Neurological Institute, Korea University Guro Hospital, Korea University Medicine, 148 Gurodong-ro, Guro-gu, Seoul, 08308, Republic of Korea; 9 Graduate School of Medical Science and Engineering, KAIST, 291 Daehak-ro, Yuseong-gu, Daejeon, 34141, Republic of Korea

**Keywords:** EDB-Fibronectin, Glioma, Big Data, Biomarkers, Micelles

## Abstract

Extra-domain B of fibronectin (EDB-FN) is an alternatively spliced form of fibronectin with high expression in the extracellular matrix of neovascularized tissues and malignant cancer cells. In this study, we evaluated the practicality of using EDB-FN as a biomarker and therapeutic target for malignant gliomas (MGs), representative intractable diseases involving brain tumors.

**Methods:** The microarray- and sequence-based patient transcriptomic database 'Oncopression' and tissue microarray of MG patient tissue samples were analyzed. EDB-FN data were extracted and evaluated from 23,344 patient samples of 17 types of cancer to assess its effectiveness and selectivity as a molecular target. To strengthen the results of the patient data analysis, the utility of EDB-FN as a molecular marker and target for MG was verified using active EDB-FN-targeting ultrasmall lipidic micellar nanoparticles (~12 nm), which had a high drug-loading capacity and were efficiently internalized by MG cells *in vitro* and *in vivo*.

**Results:** Brain tumors had a 1.42-fold cancer-to-normal ratio (*p* < 0.0001), the second highest among 17 cancers after head and neck cancer. Patient tissue microarray analysis showed that the EDB-FN high-expression group had a 5.5-fold higher risk of progression than the EDB-FN low-expression group (*p* < 0.03). By labeling docetaxel-containing ultrasmall micelles with a bipodal aptide targeting EDB-FN (termed APT_EDB_-DSPE-DTX), we generated micelles that could specifically bind to MG cells, leading to superior antitumor efficacy of EDB-FN-targeting nanoparticles compared to nontargeting controls.

**Conclusions:** Taken together, these results show that EDB-FN can be an effective drug delivery target and biomarker for MG.

## Introduction

Malignant glioma (MG) is one of the most dreaded tumors, having no cure despite aggressive treatments. Glioblastoma multiforme (GBM), the most malignant and common type of glioma, has a median survival time of less than two years even with surgical and medical interventions 1, 2. In addition to the highly proliferative and invasive features of MG, cellular heterogeneity along with the blood-brain barrier and blood-brain tumor barrier (BBTB) hinder its treatment 3, 4. To overcome these limitations, molecular and genetic studies and rearrangement of brain tumor classifications have become more crucial; the importance of these approaches has guided the incorporation of molecular biology into the pathological classification and revamping of diagnostic and therapeutic planning strategies.

Representative phenotypic-genotypic diagnostic markers that are currently implemented include O^6^-methylguanine-deoxyribonucleic acid (DNA) methyltransferase promoter (MGMT) methylation, isocitrate dehydrogenase-1 (IDH-1) mutation, and chromosomal 1p/19q deletion, and the assessment of their status is profoundly affecting personalized treatment decisions and prognostic predictions 5, 6. In addition, many other biomarkers, such as alpha-thalassemia/mental retardation syndrome X-linked and telomerase reverse transcriptase promoter mutations, are still undergoing validation, and their effectiveness is actively being verified 7. However, most biomarkers evaluated and verified in MG to date have not yet been developed as therapeutic targets; only their usefulness for assessing drug acceptability or patient survival has been confirmed. Therefore, the discovery of biomarkers that can provide effective diagnostic and therapeutic import continues to be in demand.

Fibronectin is a glycoprotein found primarily in the extracellular matrix and plasma membrane. It regulates cell migration and adhesion while binding a variety of extracellular matrix proteins, such as integrin, collagen, and fibrin. Fibronectin monomers are classified into three types by their repeating units—type I, II, and III. Alternative splicing domains generated at three regions of the fibronectin gene generate several splicing patterns. The resulting isoforms are named according to the splice site location in the type III repeat unit: fibronectin with extra-domain A (EDA-FN), extra-domain B (EDB-FN), and the type III connecting segment (IIICS-FN) 8, 9. EDB-FN is an oncofetal antigen. EDB-FN overexpression occurs in various human cancers, such as non-small cell lung carcinoma, Hodgkin lymphoma, and prostate cancer 10-12. In addition, EDB-FN acts as an angiogenesis marker in head and neck cancer 13. Regarding the role of fibronectins in the brain, EDB-FN is suggested for use as a tracing tool to diagnose primary GBM in patients 14 and as a novel target for GBM treatment, glioma radioimmunotherapy, in a rodent model 15, 16. EDB-FN is considered a differentiated fibronectin isoform that can act as a cancer-specific marker and target 17, 18. Although research on EDB-FN is actively being conducted, its roles are still being unveiled.

In this study, we investigated EDB-FN expression in MG and the relationship between EDB-FN and patient prognosis through patient sample-based big data analysis and tissue microarray. Moreover, to validate the proof-of-concept for the diagnostic and therapeutic utility of EDB-FN as a biomarker and a target in MG, *in vitro* and *in vivo* experiments were conducted following the development of an ultrasmall micellar lipidic nano-drug delivery system (DDS) specifically targeting EDB-FN.

## Methods

### Gene expression profiles and survival analysis

All gene expression profiles were downloaded from Oncopression 19, and data related to EDB-FN were collected by searching for fibronectin 1 (FN1, Entrez Gene ID: 2335), also known as FN or ED-B. Variants of the FN1 gene were investigated using ClinVar (https://www.ncbi.nlm.nih.gov/clinvar), and 126 variants were identified. Because the transcriptomic database analysis was aimed at screening expression levels in MG, analysis of the variants was not performed in this study. The transcriptomic expression levels of EDB-FN were normalized by Single Channel Array Normalization and Universal exPression Codes (SCAN.UPC) package of R 20 and presented as UPC values. More specifically, a single-sample normalization method 21 was used for Oncopression data and all samples were taken from the same Affymetrix Human Genome U133 Plus 2.0 (GPL570 or A-AFFY-44) platform. The expression values ranged from 0.0 to 1.0, where 1.0 indicates full transcriptional activation. The 'cancer-to-normal' ratio was calculated by dividing the cancer expression values by the mean normal expression values. For survival analysis, we collected brain tumor datasets containing expression profiles and patient prognostic information. After completing data collection, groups with fewer than 30 samples were excluded from the analysis. All gene expression values were quantile normalized by datasets. Z-values calculated from a log-rank test for each dataset were averaged by the Lipták method using the square root of the patient number of each dataset as the weight 22, 23.

### Patient tissue microarray (TMA) sample preparation and interpretation

Patient tissue samples donated and preserved in paraffin blocks after surgery and pathologic diagnosis were used from 21 adults aged 18 to 75 years who were diagnosed with GBM. TMA slides consisting of a total of 65 tissue samples from 3 to 4 different tumor sites per patient were prepared. Tissue cores 2 mm in diameter were carefully transferred to recipient paraffin blocks containing 45 holes per block. The filled recipient blocks were embedded in paraffin and 3 µm-thick sections were cut and mounted on slides. Tissue staining was followed by the method described below in the immunohistochemistry section. After immunostaining, we received readings from a pathologist on the level and presence of EDB-FN through a blind review. Staining intensity was scored from none or '1+' (very weak positive) to '4+' (very strong positive). Results of '1+' and '2+' were classified as low-expression group, and '3+' and '4+' were classified as high-expression group. The results of the EDB-FN expression level in TMA patient tissues were linked to each patient's clinical data in order to analyze the correlation between the expression and the patient's prognosis. Patient prognosis was analyzed using overall survival and progression free survival (PFS) as variables.

### Materials

Polyethylene glycol (_2000_)-1,2-distearoyl-*sn*-glycero-3-phosphoethanolamine (ammonium salt) (PEG_2000_-DSPE), DSPE-N-(lissamine rhodamine B sulfonyl) (ammonium salt) (Rh-DSPE), and N-maleimide-PEG_2000_-DSPE (ammonium salt) (Mal-PEG_2000_-DSPE) were purchased from Avanti Polar Lipids (CA, USA). The EDB-FN-specific peptide *N'*-CSSPIQGSWTWENGK(**C**)WTWGIIRLEQ-*C'* was a custom-synthesized peptide ordered from Anygen Corp. (Gwangju, Republic of Korea). The mouse anti-EDB-FN antibody and anti-mouse fluorescein isothiocyanate (FITC)-conjugated secondary antibody were purchased from Abcam (Cambridge, UK). Docetaxel (DTX) and Sepharose CL-4B columns were purchased from Sigma-Aldrich (MO, USA), mounting solution was purchased from Dako Diagnostics (Glostrup, Denmark), and an Alamar Blue assay kit was purchased from Thermo Fisher Scientific (MA, USA). All reagents were laboratory grade and were used as received.

### Cell culture

U87MG, U251MG, U373MG, MCF7, PC3, B16F10, and B16F1 cells were purchased from the American Type Culture Collection (VA, USA). All cells were maintained at 37 °C in a humidified 5% CO_2_ environment. Cells were cultured in minimal essential medium (MCF7), RPMI (PC3, B16F10, B16F1), or Dulbecco's modified Eagle's medium (U87MG, U251MG, U373MG) supplemented with 10% fetal bovine serum (FBS; Gibco, IL, USA), 100 U/mL penicillin (Gibco), and 100 µg/mL streptomycin (Gibco). All cell culture media were purchased from Gibco.

### In vitro 3D spheroid culture

For the 3D spheroid culture, all 3 MG cell lines (U87MG, U251MG, and U373MG) were seeded into a Nunclon Sphera Microplate 96-well round bottom plate (Thermo Fisher Scientific) at 1-5 × 10^3^ cells per well. The plate was then centrifuged at 200 x g for 2 min before being placed in a 37 °C, 5% CO_2_ incubator. The cells were incubated for 6 days, and half of the medium was changed on the third day. For imaging, the formed spheroids were transferred to an 8-well chambered coverglass slide (Thermo Fisher Scientific), fixed with 4% (w/v) paraformaldehyde (Wako, VA, USA), and immunocytochemistry was performed as described in the staining section below.

### In vivo MG flank xenograft model for EDB-FN immunohistochemistry

U87MG, U251MG, and U373MG cells were injected into the right flank of BALB/c nude mice at 5 × 10^6^ cells per mouse to create flank subcutaneous xenograft mouse models (n = 1 mouse per group). Once the tumors reached a volume of 80-120 mm^3^, mice were sacrificed and immunocytochemistry of the excised tumors was performed as described in the staining section below.

### Real-time quantitative reverse transcription-polymerase chain reaction

Cells were collected, and ribonucleic acid (RNA) was extracted with RiboEx using an RNA isolation kit (GeneAll, Seoul, Republic of Korea). Complementary DNA (cDNA) was synthesized by reverse transcription using 1 µg of total RNA from each sample. The following genes were evaluated: the EDB-FN gene (forward primer: 5'-AACTCACTGACCTAAGCTTT -3'; reverse primer: 5'-CGTTTGTTGTGTCAGTGTAG-3'); and the glyceraldehyde 3-phosphate dehydrogenase gene (forward primer: 5'-AATCCCATCACCATCTTCCA- 3′; reverse primer: 5'-TGGACTCCACGACGTACTCA-3'). The polymerase chain reaction protocol consisted of an initial denaturation step at 94 °C for 5 min; 30 cycles at 94 °C for 1 min (denaturation), 55 °C for 1 min (annealing), and 72 °C for 2 min (extension); and a final extension step at 72 °C for 7 min 24. One microgram of cDNA was added to 4 µL of ultrapure water, and 5 µL of SYBR Green real-time mixture (Takara, Tokyo, Japan) was added prior to running the real-time polymerase chain reaction (Qiagen, Tokyo, Japan). The messenger RNA (mRNA) level of each gene was quantified using the 2^-ΔΔCt^ method and normalized to that of glyceraldehyde 3-phosphate dehydrogenase.

### Cell and Tissue Staining

#### Immunocytochemistry

For 2D staining of 7 different cell lines, 10,000 cells were seeded on an 8-chambered cover glass slide 24 h before staining. For 3D staining, spheroids were moved from the cultured dish to the 8-well chambered coverglass slide. Incubated cells were rinsed three times with cold phosphate buffered saline (PBS; Welgene, Gyeongsan, Republic of Korea), fixed with 4% (w/v) paraformaldehyde for 15 min at room temperature, washed with PBS, permeabilized with 0.1% (v/v) Triton X-100 (Amresco, PA, USA)/PBS for 10 min, and incubated with primary antibody against EDB-FN (ab154210, 1:500 for 2D, 1:100 for 3D; Abcam) in 1% (w/v) bovine serum albumin (BSA; Millipore, MA, USA)/0.1% (v/v) Triton X-100/PBS at 4 °C overnight. After washing with PBS, immuno-labeled proteins were visualized by treatment with fluorescence-conjugated secondary antibodies for 60 min at room temperature. Cells were washed with PBS, mounted with 4',6-diamidino-2-phenylindole (DAPI) mounting medium (Vector laboratories, CA, USA), sealed with cover slips, and examined using a confocal laser scanning microscope (LSM 700; Carl Zeiss, NY, USA).

#### Immunohistochemistry of paraffin-embedded TMA and animal samples

The staining status of EDB-FN was analyzed by applying tissue microarray to patient tissue samples and immunohistochemistry to subcutaneous xenograft animal tissue samples. The TMA slides were dewaxed by heating at 55 °C for 30 min followed by three 5 min-washes with xylene and rehydration by 5 min-washes with 100, 95, and 80% (v/v) ethanol serially up to pure distilled water. Antigen retrieval was obtained by heating the sections at 95 °C for 30 min in 10 mM sodium citrate (pH 6.0). Endogenous peroxidase activity was blocked by incubation in 3% (w/v) hydrogen peroxide for 30 min. The background reactivity was removed using a universal blocking serum (Dako Diagnostics) for 30 min at room temperature. The slides were incubated for 1 h with antibodies specific to EDB-FN (orb227981, 1:50; Biorbyt, Cambridge, UK), and then for 30 min with a biotin-labeled secondary antibody. Streptavidin-peroxidase (Dako Diagnostics) was applied and developed. After slight counterstaining with hematoxylin, the slides were dehydrated and mounted under coverslips for microscopy.

### Synthesis of the active EDB-FN-targeting micellar nano-DDS

#### Synthesis of APT_EDB_-conjugated PEG_2000_-DSPE

EDB-FN-specific aptamer-like peptide (APT_EDB_; Anygen) containing an additional cysteine residue was dissolved in dimethyl sulfoxide (Sigma-Aldrich), and Mal-PEG_2000_-DSPE was dissolved in chloroform (Sigma-Aldrich). The conjugation reaction was carried out at an APT_EDB_:Mal-PEG_2000_-DSPE molar ratio of 1:2 under inert conditions for 12 h at ambient temperature 16. APT_EDB_-conjugated PEG_2000_-DSPE (APT_EDB_-DSPE) was purified by reversed-phase high-performance liquid chromatography, and the conjugation efficiency was determined using matrix-assisted laser desorption/ionization time-of-flight mass spectrometry. To remove unconjugated peptides, dialysis was carried out using a dialysis membrane (Spectrum Chemical, NJ, USA) with a molecular weight cutoff of 3.5 kDa. After 48 h, the conjugate was lyophilized.

#### Preparation of micellar nano-DDSs with various weight percentages of APT_EDB_-DSPE

An anionic lipid film of 2 mg/mL PEG_2000_-DSPE was prepared from the stock solution. To prepare fluorescently labeled micellar nano-DDSs, Rh-DSPE labeled with 0.5 wt% rhodamine B fluorophore was added to the PEG_2000_-DSPE solution, and APT_EDB_-DSPE was added to the formulation at a concentration of 1.0, 2.5, or 5.0 wt%. For example, to formulate the active targeting APT_EDB_-DSPE micellar nano-DDS (APT_EDB_-conjugated) with 1.0 wt% APT_EDB_-DSPE, 20 µg of the APT_EDB_-DSPE conjugate was added to 2 mg/mL of the PEG_2000_-DSPE solution containing 0.5 wt% Rh-DSPE. As a negative control, a passive/nontargeting PEG_2000_-DSPE micellar nano-DDS (APT_EDB_-unconjugated) was also synthesized. All components were added to a glass vial, dried under a vacuum, and further lyophilized overnight to remove any remaining chloroform. During rehydration, 1 mL of ultrapure water (Welgene) was added to the lipid film to yield a micellar solution with a final concentration of 2 mg/mL. Rehydration was performed under constant stirring at 1,000 revolutions per min to ensure the formation of uniform-sized micelles. Using Amicon ® Ultra-15 centrifugal filter 3 kDa Units (Merck, Darmstadt, Germany), the solution in which micelles were dissolved was changed to PBS buffer. To remove oversized nanoparticles or aggregates, the solution was filtered through a 0.1-μm membrane (Millipore) and purified by size exclusion chromatography (CL-4B column; Merck). After preparation, the size and zeta potential of all micelle formulations in PBS were analyzed by dynamic light scattering (DLS) and zeta potential analysis. One milliliter of 2 mg/mL micelles was transferred into a transparent cuvette, and the hydrodynamic diameter and zeta potential of each micelle were measured using a Zetasizer Nano ZS90 (Malvern Instruments, Malvern, UK).

#### Synthesis and development of DSPE-DTX and APT_EDB_-DSPE-DTX

Passive/nontargeting DSPE-DTX (APT_EDB_-unconjugated) and active targeting APT_EDB_-DSPE-DTX (APT_EDB_-conjugated) were developed by applying the method described above and a previously developed methodology 25. First, cysteinylated APT_EDB_ was conjugated to the maleimide group on the PEG_2000_-DSPE lipid to yield the APT_EDB_-DSPE lipid. For DTX loading, DTX was dissolved in chloroform and added to the micellar lipid film at a final concentration of 50 μg/mL during rehydration. The DTX-loaded PEG_2000_-DSPE micellar nano-DDS and APT_EDB_-DSPE micellar nano-DDS, termed 'DPSE-DTX' and 'APT_EDB_-DPSE-DTX', respectively, were then filtered through a 0.1-μm membrane and purified by size exclusion chromatography.

### Validation of EDB-FN as a therapeutic target for MG using APT_EDB_-conjugated micellar nanoparticles

#### *In vitro* cellular uptake of the APT_EDB_-DSPE micellar nano-DDS

The cellular uptake efficiency of the APT_EDB_-DSPE micellar nano-DDS was determined by treating EDB-FN-positive U87MG and U251MG cells with each micelle formulation. U87MG and U251MG cells were seeded into a 96-well plate at 5,000 cells/well. After cells had grown to confluence on a sterilized coverslip, they were incubated for 1 h at 37 °C with 100 µg/mL PEG_2000_-DSPE micellar nano-DDSs or APT_EDB_-DSPE micellar nano-DDSs with an APT_EDB_-DSPE concentration of 1.0, 2.5, or 5.0 wt%. Cells were then washed with PBS, fixed with 4% (w/v) paraformaldehyde, counterstained with the nuclear dye DAPI (Invitrogen, CA, USA), mounted on glass slides, and examined to confirm the uptake rate of rhodamine B-labeled micelles by confocal laser scanning microscopy.

#### *In vitro* cytotoxicity of APT_EDB_-DSPE-DTX to U87MG and U251MG cells

To determine the value and usefulness of EDB-FN as a molecular target for MG, we encapsulated DTX into the core of PEG_2000_-DSPE and APT_EDB_-DSPE micellar nano-DDSs by the method described above. Cells were treated by coincubation with serial dilutions of each nano-DDS formulation for 24 h. After discarding the formulation, cells were further incubated for 24 h prior to the Alamar Blue assay (Bio-Rad, CA, USA). The IC_50_ value for each formulation was determined by ProBit analysis.

#### *In vivo* uptake of the APT_EDB_-DSPE micellar nano-DDS in the subcutaneous xenograft model

To evaluate the uptake of the APT_EDB_-DPSE micellar nano-DDS in an *in vivo* xenograft model, U87MG cells were injected into the right flank of BALB/c nude mice (n = 3 mice per group) at 5 × 10^6^ cells/mouse. After 3 weeks, tumor growth was measured, and the tumor volumes were determined to be 80-120 mm^3^. Then, 200 µg of the PEG_2000_-DSPE micellar nano-DDS in PBS or APT_EDB_-DSPE micellar nano-DDS in PBS was injected into each mouse, and at predetermined time points (15, 30, 60, and 120 min), the tumor uptake of rhodamine B-labeled micelles was compared using an IVIS *in vivo* imaging system (PerkinElmer, MA, USA).

#### *In vivo* antitumor efficacy of APT_EDB_-DSPE-DTX

U87MG cells were injected into the right flank of BALB/c nude mice at 5 × 10^6^ cells per mouse to create flank subcutaneous xenograft mouse models. Once the tumors reached a volume of 80-120 mm^3^, mice were treated intravenously with saline, DPSE-DTX in PBS, or APT_EDB_-DSPE-DTX in PBS at a DTX concentration of 5 mg/kg (n = 5 mice per group, including n = 1 mouse per group for representative tumor tissue excision). Based on previous research protocols 26, each formulation was administered intravenously three times every other day for treatment. The tumor size was measured every three other days until excision. The tumor volume was calculated by the following formula: (length x width x height) x 0.5. The percentage of tumor inhibition was calculated by the following formula: [ 1 - { (T_DayE_ - T_Day1_) / T_Day1_ × C_Day1_ / (C_DayE_ - C_Day1_) } ] × 100 (T_DayE_ = tumor volume of treatment group at the end of the experiment; T_Day1_ = tumor volume of treatment group at Day 1; C_DayE_ = tumor volume of control group at the end of the experiment; C_Day1_ = tumor volume of control group at Day 1).

To create orthotopic xenograft mouse models, the heads of BALB/c nude mice were fixed using a stereotactic device, and a small burr hole was made with a high-speed drill at 2 mm lateral to the bregma and 1 mm anterior to the coronal suture according to the method used by Ozawa and James 27. U87MG cells were injected at a depth of 3 mm from the inner cortical bone of the skull using a 22-gauge needle (Hamilton Company, NV, USA) at 3 × 10^5^ cells per mouse. After 7 days, the mice were treated with saline, DSPE-DTX, or APT_EDB_-DSPE-DTX at a concentration of 10 mg/kg DTX once intravenously (n = 4 mice per group). On the 21st day after transplantation, mice were sacrificed for tumor size analysis. After perfusion with 10% (v/v) formalin (Sigma-Aldrich), each brain was removed and embedded in an optimal cutting temperature compound (Sakura Finetek, Tokyo, Japan). The embedded brain samples were frozen and sliced into 20 μm by cryostat sectioning. The brain slices were stained using a hematoxylin and eosin (H&E) staining kit (ScyTek, UT, USA), and tumor volume was calculated by the following formula: (length x width x width) x 0.5. The longest dimension was set as the length, and the longest perpendicular diameter in the same plane was set as the width.

### Imaging and statistical analysis

Image processing and data analysis were conducted using ImageJ software (http://rsb.info.nih.gov/ij/). All data were analyzed using GraphPad Prism 7 software (GraphPad, CA, USA) and are presented as the means ± standard deviations (std. devs.), except IC_50_ values, which are presented as the means ± standard errors (std. errors). Comparisons between groups were conducted primarily with an unpaired two-tailed *t* test with Welch's correction (Welch's *t* test) for normally distributed data and with the Mann-Whitney test for non-normally distributed data. *P* values of < 0.05 were considered statistically significant and are shown as *p* < 0.05 (*), *p* < 0.01 (**), *p* < 0.001 (***), and *p* < 0.0001 (****).

### Ethical approval

All applicable international, national, and/or institutional guidelines for the care and use of animals were followed. All procedures performed in studies involving animals were in accordance with the ethical standards of the KAIST Institutional Animal Care and Use Committee and of the Korea University College of Medicine (IACUC No. KA2013-13 and KOREA-2019-0123). The patient sample study adhered to the guidelines and protocols approved by the Korea University Guro Hospital Institutional Review Board (IRB No. 2017GR0330).

## Results

We analyzed normalized big data and patient tissue samples of EDB-FN expression levels to evaluate the feasibility of using EDB-FN as a marker and target among various cancers. In addition, we examined the usefulness of EDB-FN as a prognostic marker and as a drug delivery target in MG, one of the cancers with the highest cancer-to-normal EDB-FN expression ratio.

### EDB-FN expression in human brain pathologies

#### EDB-FN expression in various organs and cancers

**Figure 1:**
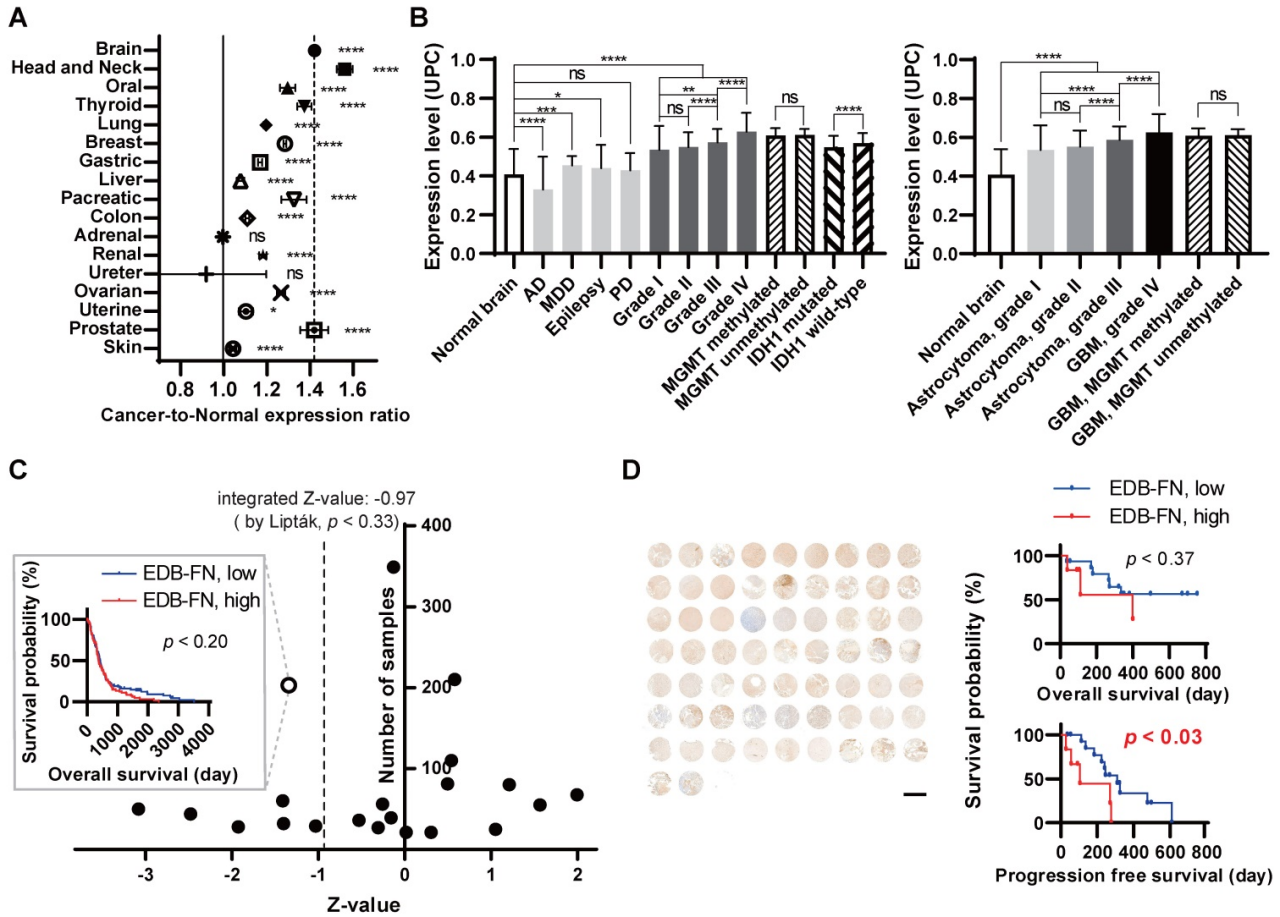
** Extra-domain B of fibronectin (EDB-FN) expression in human brain pathologies.** Patient sample-based transcriptomic database 'Oncopression' analysis. **(A)** The 'cancer-to-normal' ratio of EDB-FN expression in the 17 major organs. Total of 18,850 cancer samples and 4,494 normal samples were analyzed. The Mann-Whitney test was used for statistical analysis. The data are presented as the means ± 95% confidence interval. **(B)** EDB-FN expression in various brain pathologies, including malignant glioma (MG). Total of 3,687 samples of brain pathologies were analyzed. Expression levels of the other brain pathologies and of the brain tumor grades were compared with the value of normal brain. The Mann-Whitney test was used for statistical analysis. The results are presented as Universal exPression Codes (UPC) value.** (C)** Relationship between EDB-FN expression and prognosis of GBM patients. Total of 1,615 samples from 21 glioblastoma multiforme datasets were analyzed. The negative Z-value indicates a worse prognosis. The integrated Z-value was obtained by using Lipták's method. The black circles indicate the Z-value of each dataset, and a Kaplan-Meier survival curve was generated from the representative PMID-18772890-TCGA dataset (empty circle in the graph), which had the largest sample number and a Z-value of -1.34. **(D)** Correlation between EDB-FN expression and survival in GBM patients. Twenty-one patients with GBM were analyzed. Survival analysis was performed by integrating the patient's clinical data and EDB-FN expression level. TMA scale bar = 2 mm. Kaplan-Meier survival curve was generated. *p < 0.05, **p < 0.01, ***p < 0.001, ****p < 0.0001, ns: not statistically significant. AD: Alzheimer's dementia; EDB-FN: extra-domain B of fibronectin; GBM: glioblastoma multiforme; IDH-1: isocitrate dehydrogenase-1; MDD: major depressive disorder; MGMT: O^6^-methylguanine-deoxyribonucleic acid methyltransferase promoter; PD: Parkinson's disease; UPC: Universal exPression Codes value.

To verify the degree of EDB-FN expression in normal and cancer patient tissues, cancer-to-normal comparisons of EDB-FN in various organs and in brain tumors were performed using Oncopression. EDB-FN expression levels in 18,850 samples of a total of 17 cancers originating from various organs and 4,494 normal samples of each organ were compared and analyzed. Fifteen cancers showed a statistically significant difference in EDB-FN expression in cancer vs. normal tissues; however, no difference was found for adrenal and ureter cancers (Figure 1A, Table 1). Among the cancers, head and neck cancer and brain tumors exhibited the highest increase in the cancer-to-normal ratio of EDB-FN expression, approximately more than 1.42 (*p* < 0.001), which was high compared to that in other cancers.

#### EDB-FN expression in human brain pathologies and brain tumors

To specifically analyze EDB-FN expression in the brain, we analyzed a total of 3,687 samples of various brain pathologies, including brain tumors, according to the grade and molecular biological status (Figure 1B, Table 2). Analysis of nontumor brain pathologies indicated no significant difference in EDB-FN expression between tissues of Parkinson's disease patients compared to normal brain tissues. However, a significant decrease in EDB-FN expression was observed in tissues of patients with Alzheimer's dementia (*p* < 0.0001), and an increase was observed in tissues of patients with major depressive disorder (*p* < 0.001) and epilepsy (*p* < 0.05).

In brain tumor tissues, EDB-FN expression was universally increased regardless of grade or molecular status compared to normal tissues, with a *p* value of < 0.0001. Grade IV brain tumors showed the highest EDB-FN expression levels (an increase of approximately 1.5-fold relative to normal tissues). EDB-FN expression levels increased as grades progressed from I to IV (grade I, 1.3-fold; grade II, 1.3-fold; grade III, 1.4-fold; grade IV, 1.5-fold). There was no significant difference in EDB-FN expression in grades I and II, but there was a significant difference between grades II and III (*p* < 0.0001), as well as between grades III and IV (*p* < 0.0001). In the analysis of MGMT and IDH-1, which are representative molecular biomarkers of brain tumors, EDB-FN expression between the MGMT methylated group and MGMT unmethylated group did not show any significant difference (both 1.5-fold). However, although the fold difference between the two groups was small, EDB-FN expression in the IDH-1 wild-type group, which is known to have a worse prognosis, was significantly higher than in the IDH-1 mutated group (IDH-1 mutated, 1.3-fold; IDH-1 wild-type, 1.4-fold, *p* < 0.0001). These results suggest that EDB-FN can be a useful target for brain tumors, especially MG, regardless of the molecular status.

Furthermore, the analysis of the expression of EDB-FN in brain tumors was analyzed in detail as a typical brain tumor type, astrocytoma. Except that the statistical difference between grade I astrocytoma and grade III astrocytoma was enhanced from *p* < 0.01 to *p* < 0.0001, EDB-FN expression in astrocytoma showed similar trends and differences with the results of analyzing EDB-FN expression by grade for all brain tumors. IDH-1 comparative analysis in astrocytoma was excluded because it did not meet the analysis conditions requiring there to be 30 or more samples in the group.

#### EDB-FN as a prognostic biomarker for GBM

As an additional assessment of clinical significance, the applicability of EDB-FN as a prognostic biomarker in brain tumors, especially in the GBM group with the highest EDB-FN expression levels ('grade IV'), was analyzed using quantile normalization values from the datasets. For prognostic analysis, EDB-FN expression values in a total of 1,615 samples from 21 GBM datasets were classified into high- and low-expression groups based on the median expression values, and overall survival analysis was performed (Figure 1C, Table S1). The integrated Z-value of EDB-FN expression in GBM was -0.97 (*p* < 0.33), indicating that high EDB-FN expression in GBM patients may point to worse overall survival prognoses but have no statistically significant value (Figure 1C).

We performed TMA analysis using patient samples for in-depth and direct confirmation of *in silico* data. The expression of EDB-FN in GBM tissues and the overall survival and progression free survival of patients corresponding to each tissue were analyzed (Figure 1D). We used tissue samples from a total of 21 patients, 13 males and 8 females with a mean age of 51.0 years, who were diagnosed pathologically with GBM. Positive staining results in all 21 patients and 98.5% of samples from the patients (64 out of 65; negative staining, 1 sample, 1.5%; '1+', 15 samples, 23.1%; '2+', 17 samples, 26.2%, '3+', 28 samples, 43.1%; '4+', 4 samples, 6.2%) confirmed that EDB-FN may be useful as a cancer diagnostic biomarker in GBM. To summarize by reclassifying the EDB-FN low-expression group and high-expression group as the mean value of the staining results for each patient, the low-expression group was 71.4% (15 of 21 patients) and the high-expression group was 28.6% (6 of 21 patients). The difference between overall survival and progression free survival was analyzed between the two groups. The difference in overall survival between the EDB-FN low- and high-expression groups was not as statistically significant (*p* < 0.36; hazard ratio = 2.12; 95% confidence interval = 0.42-10.78) as *in silico* Oncopression analysis result. However, the median of progression free survival between the groups was significantly different (*p* < 0.03; low-expression, 310.0 days; high-expression, 105.0 days). The EDB-FN high-expression group had a more than 5.5-fold increased risk of progression compared to the low-expression group (hazard ratio = 5.53; 95% confidence interval = 1.22-25.05).

### EDB-FN expression in cancer cell lines

**Figure 2:**
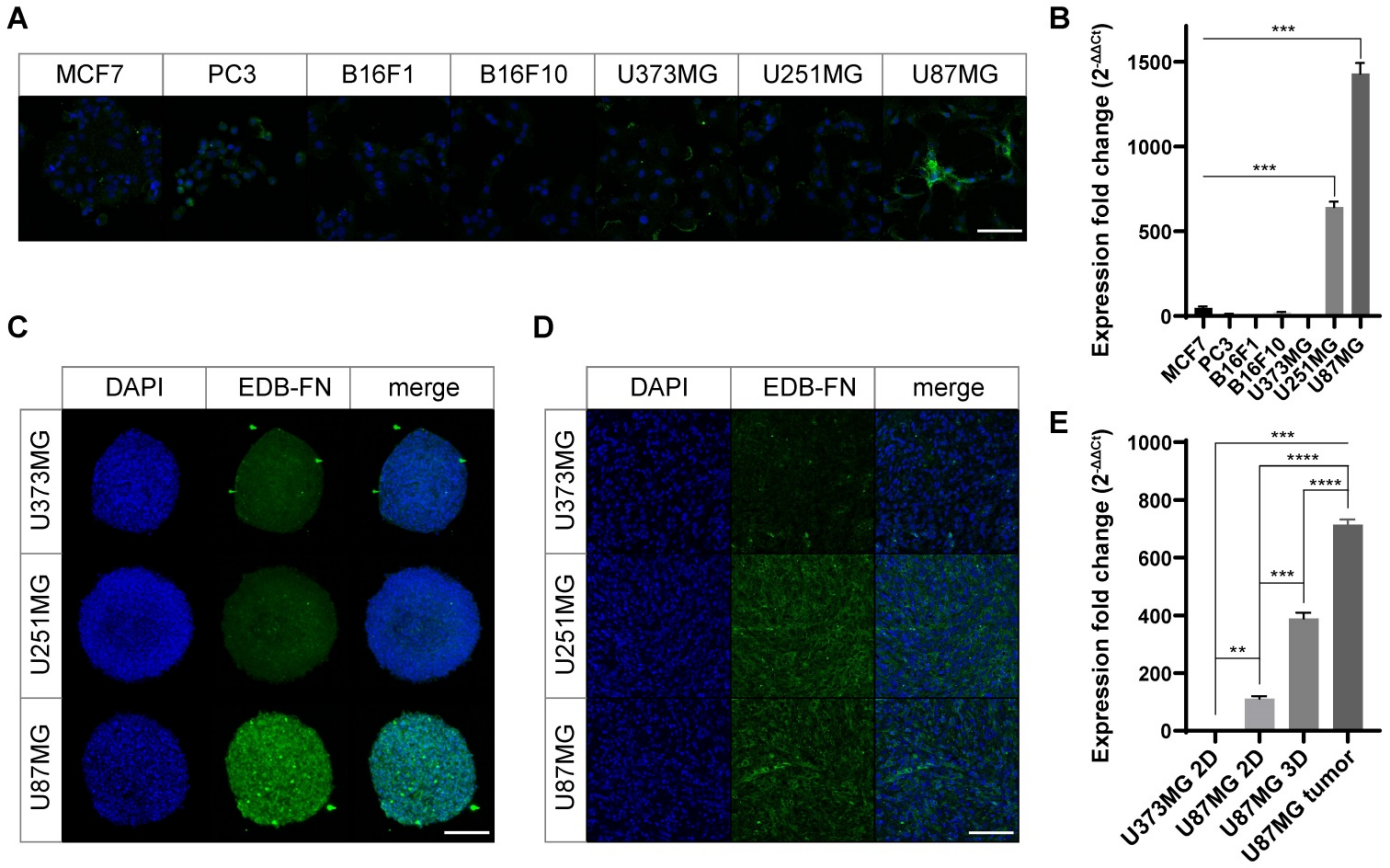
** Overexpression of EDB-FN in MG cells.** To verify the expression level of EDB-FN. **(A)** EDB-FN expression pattern in 2D cultures of various cancer cell lines (scale bar = 100 µm). Green represents EDB-FN; Blue represents 4',6-diamidino-2-phenylindole (DAPI). **(B)** EDB-FN mRNA expression analysis using quantitative real-time PCR (qRT-PCR) after extracting total RNA from each 2D cultured cell line of various cancers. 2^-∆∆Ct^ was used and glyceraldehyde 3-phosphate dehydrogenase was set as the inner control. Confirmation of EDB-FN expression pattern through immunofluorescence staining in 3D culture (scale bar = 200 µm) **(C)** and subcutaneous transplanted cancer tissues (scale bar = 100 µm) **(D)** of MG cell lines. **(E)** qRT-PCR analysis using total RNA extracted from U373MG cells (2D monolayer culture) or U87MG cells (2D monolayer culture, 3D spheroid, and subcutaneous tumor tissue). Statistical analysis: Welch's *t* test. **p < 0.01, ***p < 0.001, ****p < 0.0001, ns: not statistically significant. The results are presented as the means ± standard deviations of quadruplet determinations. EDB-FN: extra-domain B of fibronectin; MG: malignant glioma.

We screened various human cancer cell lines to determine their EDB-FN expression levels. The breast cancer cell line MCF7, prostate cancer cell line PC3, melanoma cell lines B16F1 and B16F10, and MG cell lines U373MG, U87MG, and U251MG were used in experiments. The expression pattern of the EDB-FN protein was confirmed for qualitative analysis during the two-dimensional monolayer culture (Figure 2A). Weak EDB-FN expression was detected in the PC3 cell line and the U373MG cell line, but noticeable expression was observed in the U87MG cell line. In addition, qRT-PCR was performed by extracting mRNA for quantitative analysis of EDB-FN expression (Figure 2B). As a result (MCF7, 47.8 ± 7.9; PC3, 12.1 ± 2.1; B16F1, 0.6 ± 0.5; B16F10, 23.3 ± 1.4; U373MG, 2.6 ± 0.8; U251MG, 643.0 ± 31.0; U87MG, 1430.3 ± 61.4), statistically significant overexpression was observed in U251MG (*p* < 0.001, compared to all other cell lines, Welch's *t* test), and the highest expression was seen in U87MG cell line (*p* < 0.001, compared to all other cell lines, Welch's *t* test). Through these results, it was confirmed that EDB-FN was more overexpressed in MG when compared with other cancers.

To realize the tumor microenvironment, MG cell lines were cultured in 3D, and an MG flank xenograft animal model was constructed. Qualitative analysis of EDB-FN expression using immunostaining revealed that EDB-FN was more overexpressed in U87MG cell lines than in U251MG and U373MG cell lines during 3D culturing (Figure 2C). In the xenograft mouse model, the U251MG and U87MG cell lines showed relatively higher expression patterns than those of the U373MG cell lines (Figure 2D). In the quantitative analysis through mRNA qRT-PCR (Figure 2E), EDB-FN was highly expressed in the 2D cultured U87MG cell line (*p* < 0.01, Welch's *t* test) compared to the 2D cultured U373MG cell line. Compared to it, the U87MG cell line showed higher EDB-FN expression in 3D culture (*p* < 0.001, compared to 2D cultured U87MG, Welch's *t* test), which mimics the tumor microenvironment, and the highest EDB-FN expression when xenografted into animals (*p* < 0.0001, compared to 3D cultured U87MG, Welch's *t* test). These results imply a linear correlation between EDB-FN expression and tumor microenvironment similarity in MG, indicating the feasibility of using EDB-FN as a cancer biomarker for MG.

Collectively, it appears that EDB-FN is overexpressed in MG cells compared to other cancer cells. And among MG cell lines, relatively low expression of EDB-FN appears in U373MG cells that proliferate slowly 28. U87MG, which exhibits features of aggressive proliferation, exhibited the highest EDB-FN expression level, followed by U251MG, which has GBM stem-like cell characteristics 29. Based on these results, U87MG and U251MG cell lines were used in the subsequent study.

### Characteristic analysis of the synthesized APT_EDB_-DSPE micellar nano-DDS

**Figure 3:**
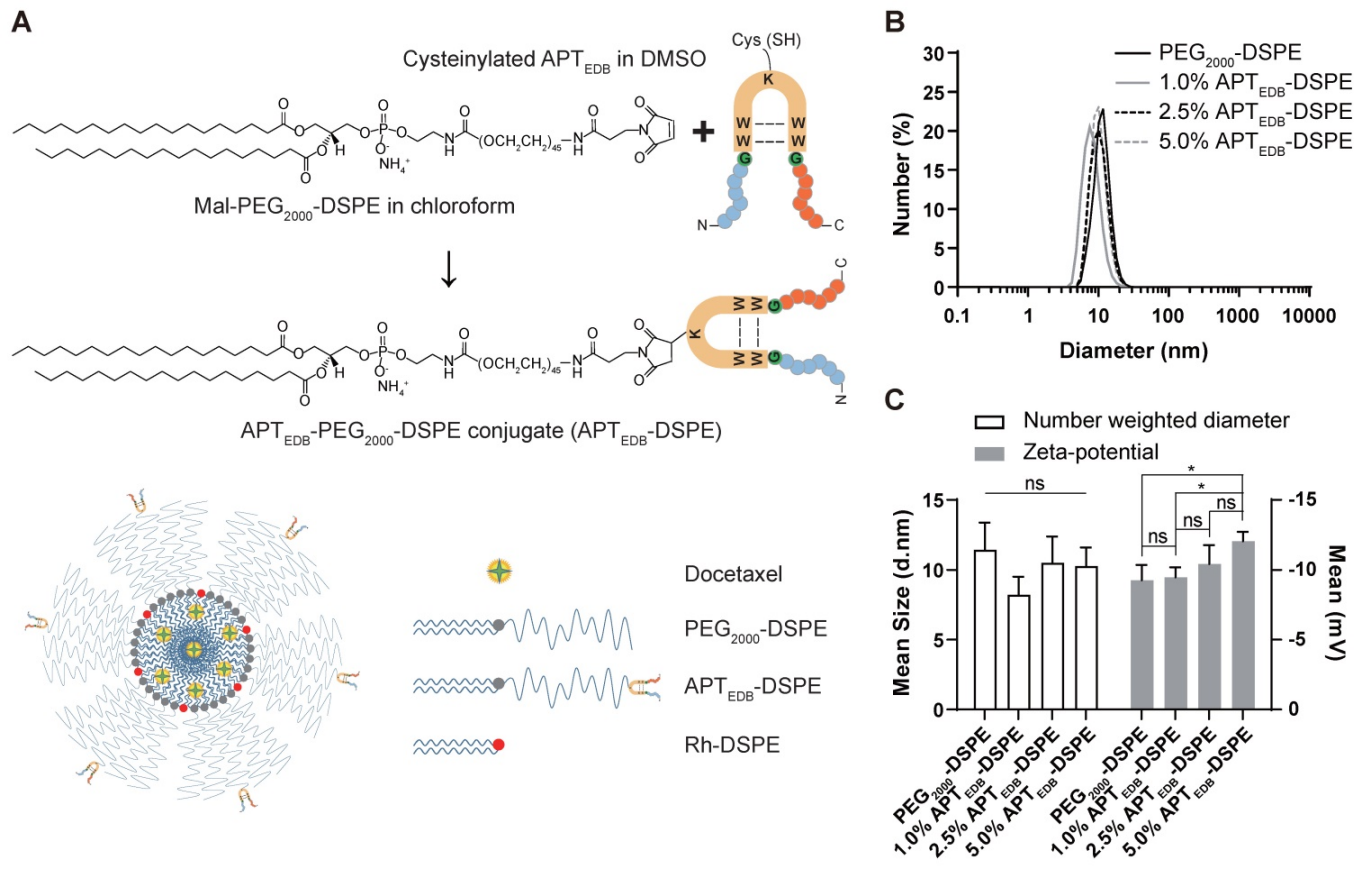
** Characteristic analysis of the synthesized APT_EDB_-DSPE micellar nano-DDS. (A)** The synthesis scheme of cysteinylated APT_EDB_ with Mal-PEG_2000_-DSPE and a schematic representation of the formulation of the APT_EDB_-DSPE micellar nano-DDS encapsulating docetaxel (APT_EDB_-DSPE-DTX). **(B)** DLS size measurement of the PEG_2000_-DSPE micellar nano-DDS (APT_EDB_-unconjugated) vs. the APT_EDB_-DSPE micellar nano-DDSs (APT_EDB_-conjugated) showed that the sizes of both types of nanoparticles were less than 12 nm (3 replicates per group). **(C)** All nanoparticle formulations had a negative zeta potential. As the APT_EDB_-DSPE concentration increased, the negative zeta potential of the nano-DDS became even more negative. Statistical analysis: Welch's *t* test. *p < 0.05, ns: not statistically significant. The results are presented as the means ± standard deviations of quadruplet determinations. DMSO: dimethyl sulfoxide; PEG_2000_-DSPE: polyethylene glycol (_2000_)-1,2-distearoyl-*sn*-glycero-3-phosphoethanolamine (ammonium salt); Rh-DSPE: DSPE-N-(lissamine rhodamine B sulfonyl) (ammonium salt).

DLS analysis of the micelles showed diameters of 11.5 ± 1.9 nm for the PEG_2000_-DSPE micellar nano-DDS and 8.2 ± 1.3 nm, 10.5 ± 1.9 nm, and 10.3 ± 1.3 nm for the APT_EDB_-DSPE micellar nano-DDS with APT_EDB_-DSPE concentrations of 1.0, 2.5, and 5.0 wt%, respectively (Figure 3A and B). The decrease in the DLS-measured size of the APT_EDB_-DSPE micellar nano-DDS compared to that of the PEG_2000_-DSPE micellar nano-DDS and the increase in the measured size of the APT_EDB_-DSPE micellar nano-DDS at APT_EDB_-DSPE concentrations of 2.5 and 5.0 wt% compared to that of the same DDS at 1.0 wt% indicate that the presence of APT_EDB_ on the outer surface of the micelles may influence conformational changes in the micellar nano-DDSs. The zeta potential, from which the surface charge of the nanoparticles can be deduced, was measured for each nano-DDS; the zeta potentials were -9.3 ± 1.1 mV for the PEG_2000_-DSPE micellar nano-DDS and -9.5 ± 0.7 mV, -10.4 ± 1.3 mV, and -12.1 ± 0.7 mV for the APT_EDB_-DSPE micellar nano-DDS with APT_EDB_-DSPE concentrations of 1.0, 2.5, and 5.0 wt%, respectively (Figure 3C).

### Cellular uptake depends on the APT_EDB_-DSPE density

**Figure 4:**
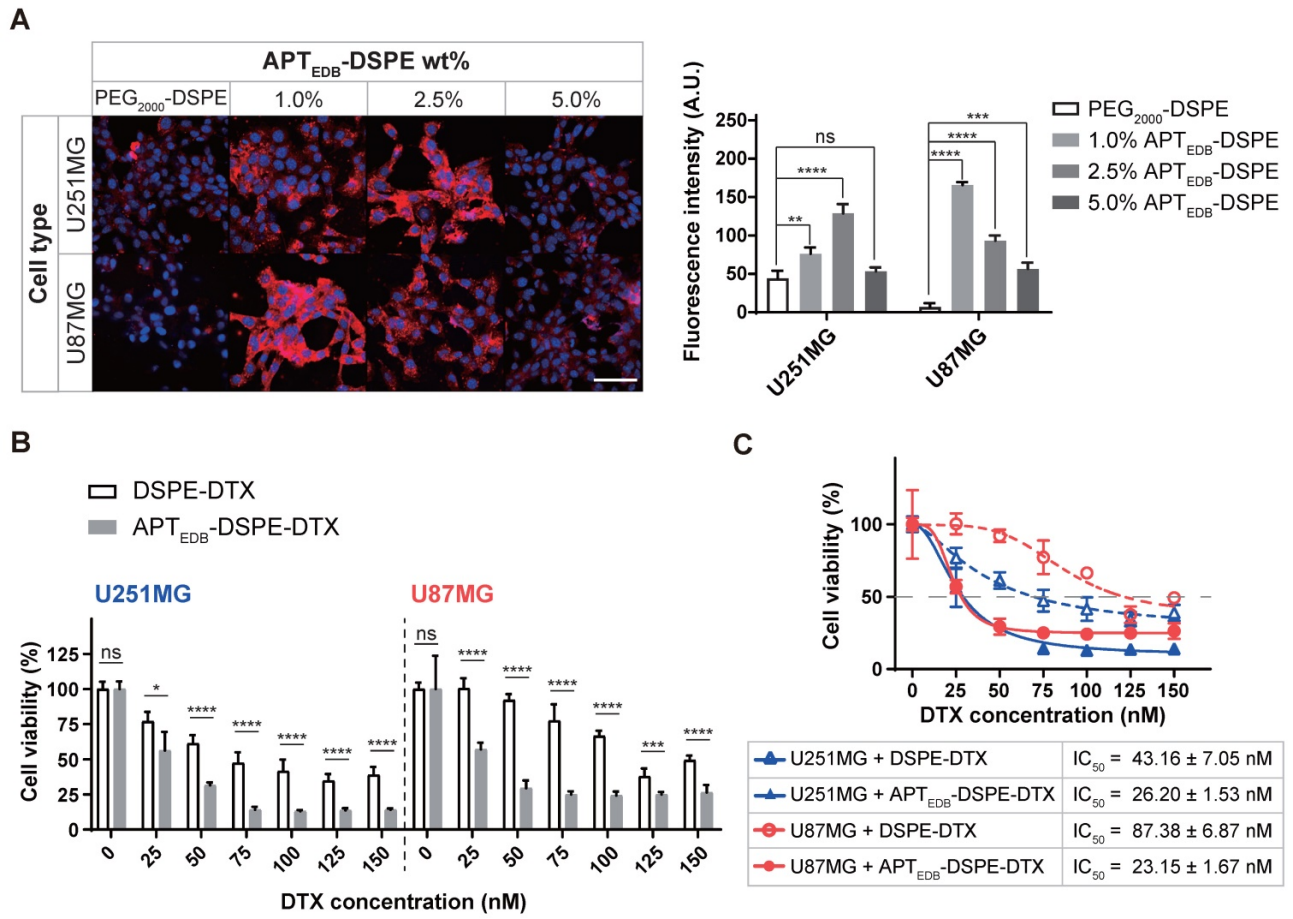
***In vitro* cellular uptake and cytotoxicity of the APT_EDB_-DSPE and APT_EDB_-DSPE-DTX micellar nano-DDS. (A)** Cell uptake of rhodamine B fluorophore-labeled nano-DDSs according to the concentration of APT_EDB_-DSPE in MG (left). Red: nano-DDSs, Blue: DAPI, Scale bar = 100 µm. Quantification analysis of the APT_EDB_-DSPE micellar nano-DDS cellular uptake via ImageJ (right). Fluorescence intensity was normalized to the DAPI signal of each cell line (quadruplet determinations). **(B)** *In vitro* cytotoxicity of DSPE-DTX and APT_EDB_-DSPE-DTX to U251MG and U87MG cells. % cell viability on the Y-axis was calculated by dividing the O.D. value under nano-DDSs treatment by the O.D. value under PBS treatment (7 replicates for DSPE-DTX and 6 replicates for APT_EDB_-DSPE-DTX). **(C)** The IC_50_ values in U87MG and U251MG cells were calculated according to the types of nanoparticles used for treatment (7 replicates for DSPE-DTX and 6 replicates for APT_EDB_-DSPE-DTX). Statistical analysis: Welch's *t* test. *p < 0.05, **p < 0.01, ***p < 0.001, ****p < 0.0001, ns: not statistically significant. The results are presented as the means ± standard deviations. A.U: arbitrary unit. APT_EDB_-DSPE: APT_EDB_-conjugated PEG_2000_-DSPE; APT_EDB_-DSPE-DTX: DTX-loaded APT_EDB_-DSPE micellar nano-DDS; DSPE-DTX: DTX-loaded PEG_2000_-DSPE micellar nano-DDS; DTX: docetaxel; PEG_2000_-DSPE: polyethylene glycol (_2000_)-DSPE (ammonium salt).

To determine the optimal targeting ligand density, APT_EDB_-DSPE was added to a chloroform solution of Rh-DSPE mixed with PEG_2000_-DSPE at concentrations of 1.0, 2.5, and 5.0 wt% prior to formation of the lipid film. Under all conditions, the uptake efficacy of the active targeting APT_EDB_-DSPE micellar nano-DDS in cancer cells was higher than that of the passive/nontargeting PEG_2000_-DSPE micellar nano-DDS (Figure 4A). Interestingly, the APT_EDB_-DSPE micellar nano-DDS with an APT_EDB_-DSPE concentration of 2.5 wt% showed the highest cellular uptake in U251MG cells (PEG_2000_-DSPE, 44.5 ± 9.6; 1.0% APT_EDB_-DSPE, 76.4 ± 8.1; 2.5% APT_EDB_-DSPE, 128.8 ± 12.1; 5.0% APT_EDB_-DSPE, 53.4 ± 5.1), whereas the APT_EDB_-DSPE micellar nano-DDS with an APT_EDB_-DSPE concentration of 1.0 wt% showed the highest cellular uptake in U87MG cells (PEG_2000_-DSPE, 6.8 ± 5.1; 1.0% APT_EDB_-DSPE, 165.7 ± 3.7; 2.5% APT_EDB_-DSPE, 93.2 ± 6.7; 5.0% APT_EDB_-DSPE, 56.7 ± 7.9). The negative charge on the nano-DDSs, which becomes more negative with increasing APT_EBD_-DSPE concentration (Figure 3C), may partially accounted for the decreased cellular uptake of the APT_EDB_-DSPE micellar nano-DDS with an APT_EDB_-DSPE concentration of 5.0 wt%. However, although both U87MG and U251MG cells are MG cancer cells, and the same targeting ligand was used, the APT_EDB_-DSPE concentration with the highest cellular uptake efficacy differed in each cell line. Thus, the ligand density might differ depending on the cell line and cell type, and in turn, the cellular uptake may vary.

To verify the APT_EDB_-DSPE micellar nano-DDS's ability to target EDB-FN, competition analysis was conducted by simultaneously treating EDB-FN high- and low-expression cells with EDB-FN-targeting aptide and APT_EDB_-DSPE micellar nano DDS. The uptake of APT_EDB_-DSPE micellar nano-DDS decreased as the concentration of EDB-FN-targeting aptide was increased in U87MG and U251MG, which are EDB-FN high-expression cells. Although the uptake with APT_EDB_-DSPE was minimal, the EDB-FN low-expression cells MCF7 and B16F1 also showed the effect of EDB-FN blocking (Figure S1A and B).

We also knocked down EDB-FN in U87MG cells to determine whether the active targeting APT_EDB_-DSPE micellar nano-DDS is dependent on EDB-FN expression. EDB-FN expression in U87MG cells was significantly reduced after treatment with EDB-FN-siRNA; while the EDB-FN expression remained unchanged in cells treated with control siRNA (control siRNA, 1.00 ± 0.08; EDB-FN siRNA, 0.45 ± 0.01; *p* < 0.01, Welch's *t* test) (Figure S2A). After treatment with these siRNAs, the uptake of APT_EDB_-DSPE micellar nano-DDS remains unchanged. However, in the cells with suppressed EDB-FN expression, the uptake of active targeting APT_EDB_-DSPE micelle nano-DDS was decreased (Figure S2B). Moreover, time-dependent uptake of APT_EDB_-DSPE micellar nano-DDS in U87 cells was also observed for 4 h. After 5 min of treatment, the intracellular APT_EDB_-DSPE micellar nano-DDS concentration gradually increased, and saturation was achieved between 1 h and 4 h. (Figure S2C).

These results indicate that APT_EDB_-DSPE micellar nano-DDS is uptaken by cells in an EDB-FN expression-dependent and time-dependent manner, and clearly show that the targeting ligand density is indeed important for the cellular uptake of nanoparticles.

### EDB-FN- targeting enhances cancer targeting and anticancer efficacy

#### *In vitro* cytotoxicity of APT_EDB_-DSPE-DTX

To determine the value and usefulness of EDB-FN as a molecular target for MG, we encapsulated DTX into the core of PEG_2000_-DSPE and APT_EDB_-DSPE micellar nano-DDSs. The loading capacity of each micellar nano-DDS was calculated to be 10 wt%, with an encapsulation efficacy of ~95%. Cell viability was evaluated using DSPE-DTX and APT_EDB_-DSPE-DTX (Figure 4B). Although the DTX in both the DSPE-DTX and APT_EDB_-DSPE-DTX systems inhibited the viability of U251MG and U87MG cells, the degree of inhibition differed. The IC_50_ values in U87MG cells were 87.38 ± 6.87 nM for DSPE-DTX and 23.15 ± 1.67 nM for APT_EDB_-DSPE-DTX, approximately 3.8-fold lower for APT_EDB_-DSPE-DTX than for DSPE-DTX (*p* < 0.0001, Welch's *t* test). The IC_50_ values in U251MG cells were 43.16 ± 7.05 nM for DSPE-DTX and 26.20 ± 1.53 nM for APT_EDB_-DSPE-DTX, approximately 1.6-fold lower for APT_EDB_-DSPE-DTX than for DSPE-DTX (*p* < 0.05, Welch's *t* test) (Figure 4C). The* in vitro* cellular toxicity data in U87MG and U251MG cells implied that superior cancer targeting and increased drug uptake may be achieved with active targeting of EDB-FN compared to passive/nontargeting. Since a significant difference in the IC_50_ values of DSPE-DTX and APT_EDB_-DSPE-DTX was observed in U87MG cells, U87MG cells were selected for *in vivo* modeling and evaluation of EDB-FN as a molecular target for MG.

#### *In vivo* uptake of the APT_EDB_-DSPE micellar nano-DDS

**Figure 5:**
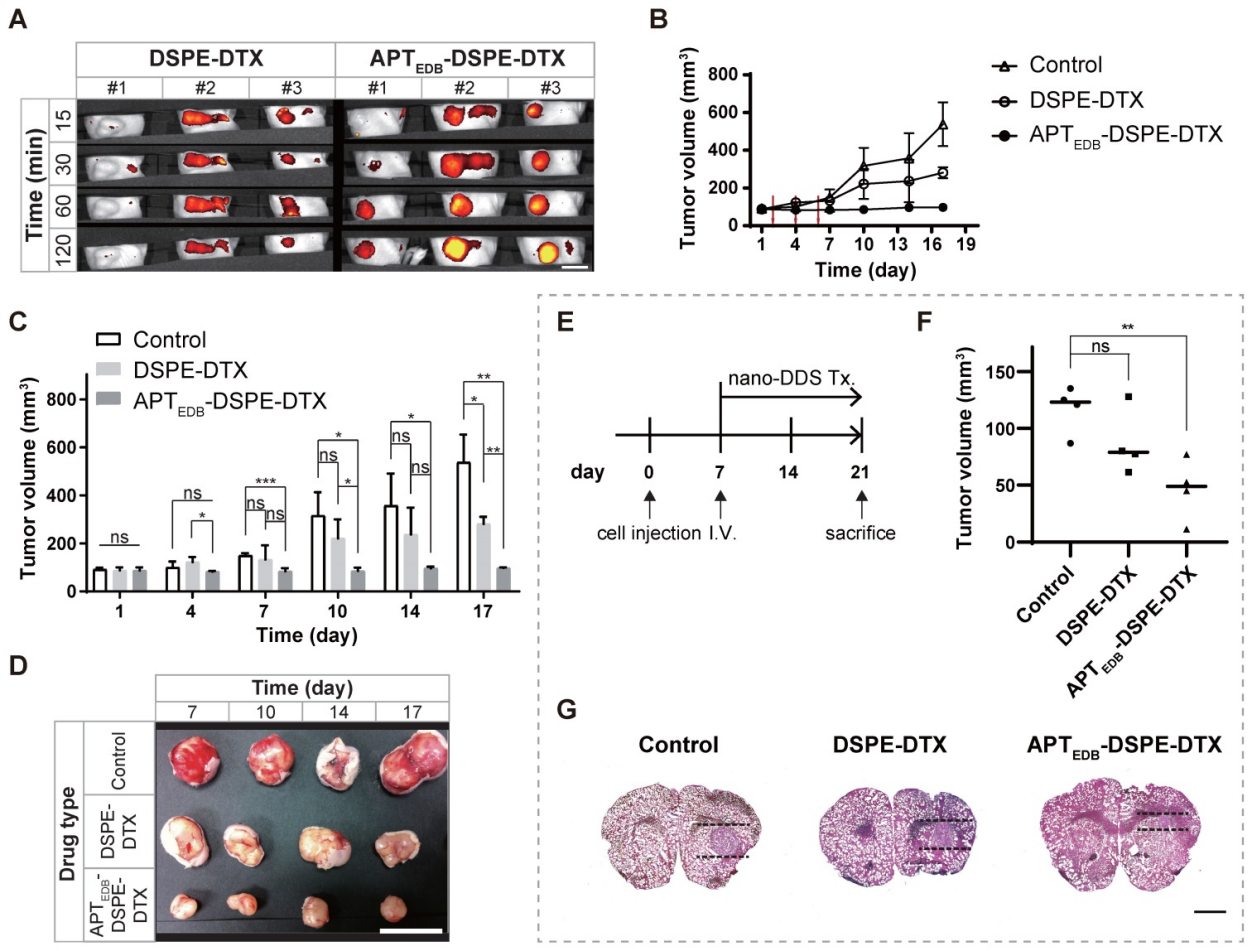
***In vivo* uptake and anticancer efficacy of APT_EDB_-DSPE-DTX.** In U87MG subcutaneous xenograft mouse model, **(A)** IVIS rhodamine B real-time imaging of PEG_2000_-DSPE micellar nano-DDS vs. APT_EDB_-DSPE micellar nano-DDS uptake in a U87MG xenograft tumor-bearing rodent model (n = 3 mice per group). Scale bar = 10 mm. **(B)** U87MG xenograft tumor growth curves according to drug treatment. The tumor size in mice was measured every 3 days (n = 4 mice per group). The red arrow indicates the drug IV infusion schedule. **(C)** Anticancer effect of micelle nano-DDSs. Changes in tumor size according to treatment with DSPE-DTX, APT_EDB_-DSPE-DTX, or saline as the negative control were compared (n = 4 mice per group). **(D)** Representative images of excised tumors from the xenograft model. Drug-treated mice were sacrificed on day 7, 10, 14, and 17 respectively (n = 1 mouse per group). Scale bar = 10 mm. In U87MG orthotopic xenograft mouse model, **(E)** Experimental schedule for orthotopic model. **(F)** Inhibitory effect of micelle nano-DDSs on malignant brain tumor (n = 4 per group). Brain slices with the largest tumor volume in all subjects were selected and analyzed. **(G)** Representative image for comparison of brain tumor size. The mouse brain was sectioned to a thickness of 20 μm, and brain tumors were identified by H&E staining. Scale bar = 1 mm. Statistical analysis: Welch's *t* test. *p < 0.05, **p < 0.01, ***p < 0.001, ****p < 0.0001, ns: not statistically significant. The results are presented as the means ± standard deviations. APT_EDB_-DSPE-DTX: docetaxel-loaded APT_EDB_-DSPE micellar nano-DDS; DDS: drug delivery system; DSPE-DTX: docetaxel-loaded PEG_2000_-DSPE micellar nano-DDS; DTX: docetaxel; nano DDS Tx: nano drug delivery system treatment.

In the real-time IVIS imaging study in the U87MG flank xenograft mouse model, the APT_EDB_-DSPE micellar nano-DDS showed a significant increase in tumor localization compared to the passive/nontargeting PEG_2000_-DSPE micellar nano-DDS; this increase in tumor localization was observed consistently from 15 min to 120 min (Figure 5A). At 15 min, minimal nanoparticle accumulation could be seen. After 30 min, the APT_EDB_-DSPE micellar nano-DDS group started to show higher accumulation. After 60 min, the APT_EDB_-DSPE micellar nano-DDS group exhibited greater accumulation of the DDS at the tumor site than the PEG_2000_-DSPE micellar nano-DDS group. In addition, tissue uptake of the micellar nano-DDSs was observed for 48 h to determine if the DDSs affect organs other than tumors (Figure S3A). The passive/nontargeting PEG_2000_-DSPE micellar nano-DDS gradually spread throughout the body within 48 h. However, the APT_EDB_-DSPE micellar nano-DDS was found to remain stable, confined to the tumor site. These results indicate the stability and high MG-targeting ability of the APT_EDB_-DSPE micellar nano-DDS. The EDB-FN-targeting ability of APT_EDB_-DSPE may have significantly increased the retention time of the APT_EDB_-DSPE micellar nano-DDS in MG by binding to EDB-FN with high affinity, which in turn increased the bioavailability of the drug in the tumor.

#### *In vivo* anticancer efficacy of APT_EDB_-DSPE-DTX

The *in vivo* anticancer efficacy was examined in the U87MG subcutaneous xenograft animal model. The efficacy was evaluated by comparing the trend of tumor growth over time in the control, DSPE-DTX, and APT_EDB_-DSPE-DTX groups (Figure 5B). The tumor volume in the control group increased approximately 5.9-fold within 16 days relative to the volume on day 1 (Day 1: 91.5 ± 7.4 mm^3^ vs. Day 17: 538.0 ± 115.9 mm^3^; *p* < 0.01, Welch's *t* test). Tumor growth was significantly inhibited by treatment with either DSPE-DTX or APT_EDB_-DSPE-DTX. By calculating the percentage of tumor inhibition as described in 'Materials and Methods', we found that DSPE-DTX inhibited tumor growth by approximately 54.8% (Day 1: 87.9 ± 12.6 mm^3^ vs. Day 17: 281.7 ± 29.4 mm^3^; *p* < 0.001, Welch's *t* test), whereas APT_EDB_-DSPE-DTX significantly inhibited tumor growth by approximately 97.6% (Day 1: 87.5 ± 12.6 mm^3^ vs. Day 17: 97.8 ± 2.6 mm^3^; *p* < 0.20, Welch's *t* test). At day 1, tumor volume did not differ significantly between groups. However, after three doses of nano-DDS injections on days 2, 4, and 6, the difference in tumor volume between groups became significant over time (Figure 5C and D). The APT_EDB_-DSPE-DTX group showed significant tumor inhibition beginning on day 7 compared to the control group (*p* < 0.001, Welch's *t* test) and beginning on day 17 compared to the DSPE-DTX group (*p* < 0.01, Welch's *t* test).

As shown in Figure S3B, no adverse event was observed in major organs such as the heart, liver, spleen, lung, and kidney due to the treatment of micelle nano-DDSs in the body, and there was no significant change in mouse weight until the end of the experiment (Day 1, control = 20.4 ± 0.6 g, DSPE-DTX = 21.4 ± 0.2 g, APT_EDB_-DSPE-DTX = 18.9 ± 0.6 g; Day 17, control = 19.8 ± 1.6 g, DSPE-DTX = 20.7 ± 1.2 g, APT_EDB_-DSPE-DTX = 19.5 ± 0.9 g) (Figure S3C). This indicates that APT_EDB_-DSPE micelle nano-DDS is biocompatible *in vivo* due to its insignificant toxicity.

#### Anticancer efficacy of APT_EDB_-DSPE-DTX in orthotopic brain tumor mouse model

To evaluate its practicality as a target for the treatment of brain tumors, we constructed a U87MG orthotopic xenograft animal model. As shown in Figure 5E, saline as a control, DSPE-DTX and APT_EDB_-DSPE-DTX were injected via intravenous injection 7 days after cell transplantation (n = 4 mice per group). After 2 weeks, the brain was extracted and the tumor volume of each group was compared. Localization of the normal and tumor areas of sliced ​​brain tissues was performed using H&E staining, and the volume of tumor was measured based on the slice in which the tumor was the largest in each individual (Figure 5F and G). As a result, we found that tumor growth was significantly inhibited by EDB-FN-targeting micelle nano-DDS treatment. Compared to the control group (116.9 ± 21.0 mm^3^), DSPE-DTX (86.7 ± 28.7 mm^3^) and APT_EDB_-DSPE-DTX (46.5 ± 27.0 mm^3^) groups had tumor growth suppression by 25.8% (*p* < 0.15, Welch's *t* test) and 60.2% (*p* < 0.01, Welch's *t* test), respectively. And although statistical significance was not shown, APT_EDB_-DSPE-DTX group showed about a 34.4% higher tumor growth suppression than DSPE-DTX group (*p* < 0.09, Welch's *t* test). No significant weight change was observed in all groups during the experiment (Day 1, control = 22.7 ± 1.3 g, DSPE-DTX = 23.3 ± 2.5 g, APT_EDB_-DSPE-DTX = 23.3 ± 1.6 g; Day 21, control = 24.5 ± 1.3 g, DSPE-DTX = 25.1 ± 1.7 g, APT_EDB_-DSPE-DTX = 25.5 ± 1.5 g) (Figure S3C). These results suggest that our EDB-FN-targeting micellar nano-DDS has the potential to treat MG and indicate that EDB-FN is a useful target for drug therapy.

In order to confirm that implanted U87MG cells in the MG orthotopic mouse models still overexpress EDB-FN, immunohistochemistry analysis was performed using mouse brain slices containing tumor in all groups. In contrast to normal area of the mouse brain tissue, EDB-FN was highly expressed in tumoral area of all groups (Figure S4A). Interestingly, EDB-FN expression within the tumor was slightly decreased in the APT_EDB_-DSPE-DTX group compared to the control and DSPE-DTX group (Figure S4B), which may suggest the possibility that APT_EDB_-DSPE-DTX had an effect on tumoral EDB-FN levels.

## Discussion

In this study, we focused on the target, EDB-FN, which is located on the surface of cancer cells and in the extracellular matrix. This is because proteins on the surface and in the extracellular matrix can provide useful targets for drug delivery systems (DDSs) as well as cancer diagnostic biomarkers 30-32. We recently established 'Oncopression', a dataset that integrates separate datasets of various cancers for comprehensive analysis 19. Among numerous specifically analyzed overexpressed markers in the dataset of patient cancer tissues, we found that EDB-FN is one of the cancer biomarkers specifically overexpressed in MG compared to other carcinomas in terms of cancer-to-normal expression levels.

Previously, we reported that EDB-FN is a useful target for multiple drug-resistant breast cancers and gliomas 33, 34. However, most previous EDB-FN studies were based only on *in vitro* and *in vivo* results, and assessments of clinical efficacy have been limited to date. Therefore, the integration of research data and clinical databases are crucial 35. In this study, we investigated the clinical relevance of EDB-FN by analyzing large quantitative datasets of messenger ribonucleic acid expression in patients with cancers spanning 17 different organs. Data extracted from cancers of various major organs and the corresponding normal tissue samples were integrated, and the differences in EDB-FN expression between each cancer tissue and its corresponding normal tissue were evaluated. For brain tumors, data for samples with various pathological statuses, such as astrocytoma, GBM, oligodendroglioma, oligoastrocytoma, ependymoma, and medulloblastoma, were included. Oligoastrocytoma has been discouraged as a diagnosis since the 2016 WHO classification was published but was included in this analysis, as we collected and analyzed samples of all brain tumors accessible through open public data. To verify the usefulness of EDB-FN in MG, the scope of the analysis was readjusted to 3,687 samples from normal brains, brains harboring nontumor diseases, and tissues from patients with diagnosed astrocytoma of any grade, including GBM, with representative molecular information, and EDB-FN was found to have significantly higher expression in all grades of brain tumors than in a normal brain. Interestingly, the results of *in silico* analysis showed that EDB-FN has higher expression than in a normal brain in other pathological conditions such as major depressive disorder and epilepsy. This result may suggest a different meaning of EDB-FN, which has been known as an oncofetal antigen to date 30, but it is considered a matter to be confirmed through additional experimental verification in order to trust this result. In addition to quantitative comparisons of EDB-FN expression, prognostic prediction was performed in GBM, a representative MG, but there was no statistically significant difference in the overall survival according to the EDB-FN expression level.

Although big data analysis using Oncopression is information extracted from a large number of patient samples, it was necessary to reinforce the evidence through more direct experimental validation of the data. In addition, there was a need to evaluate whether the expression level of EDB-FN is correlated with progression free survival, another especially important patient prognostic endpoint for GBM patients. Therefore, we newly produced TMA slides using preserved GBM patient specimens under IRB approval. The results obtained through the TMA analysis can be summarized into the following three points; 1) EDB-FN expression could be confirmed in most tissue samples of GBM patients, suggesting that EDB-FN can be considered a diagnostic biomarker for GBM; 2) There was no significant difference in overall survival between the EDB-FN low-expression group and high-expression group, which is a result of enhancing the reliability of the Oncopression analysis data; 3) There is a significant difference in progression free survival according to the EDB-FN expression level; the EDB-FN high-expression group may have a progression risk 5.5 times higher than that of the low-expression group, which suggests that EDB-FN may serve as a prognostic biomarker for GBM patients.

Taking these results into account, we speculated that EDB-FN could be used as a diagnostic and noninvasive therapeutic drug delivery target candidate in MG, and we performed conventional *in vitro* and *in vivo* experiments for validation, including orthotopic xenograft animal model experiments. Since the BBTB often exhibits minute physiologic pore sizes of less than 20 nm, which would impede the penetration of larger DDSs, using much smaller sized nano-DDSs would ensure easy penetration through the BBTB 36, 37. Therefore, we worked to improve our drug delivery strategy and developed an ultrasmall micelle (~12 nm) using a DSPE polymer and attached an APT_EDB_ to the surface of the micelle, constructing a system that could be used for the diagnosis and treatment of MG. The size of the drug delivery system was greatly reduced from the previously used approximately 115-118 nm sized liposomes or 34 nm sized superparamagnetic iron oxide nanoparticles 33, 34, 38 to the current sub-12 nm sized micelles (Figure 3).

**Figure 6:**
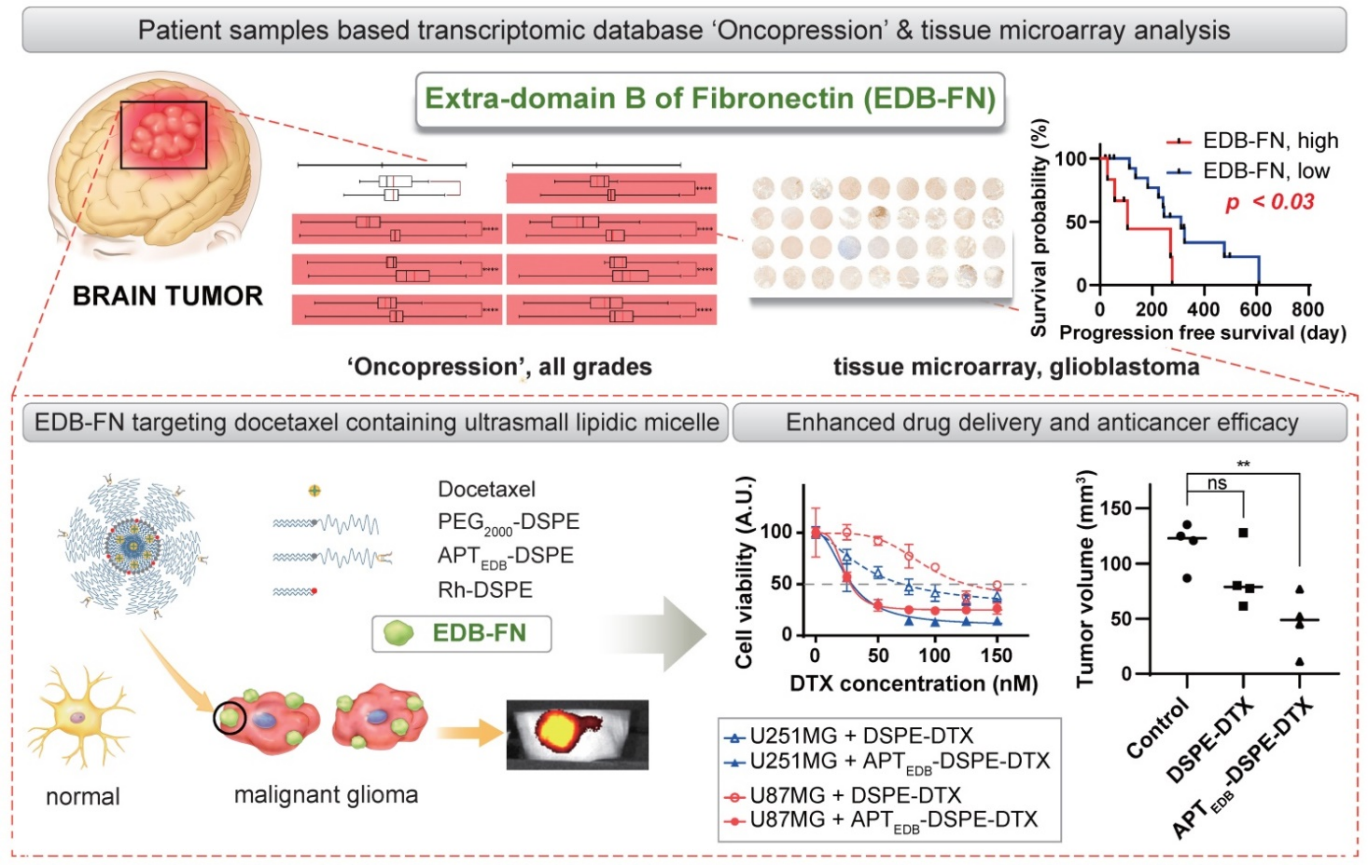
Schematic illustration of the clinical significance of EDB-FN as a potential biomarker and the feasibility of using EDB-FN as a targeting ligand for MG.

We quantitatively compared the expression level of EDB-FN and the patient prognostic relationship (Figure 1 and 2) and examined the usefulness of EDB-FN-targeting in MG (Figure 4 and 5). The overexpression of EDB-FN and the enhanced drug delivery by EDB-FN-targeting DDSs were shown not only in monolayer cell cultures but also in orthotopic xenograft models, which may confirm that EDB-FN is expressed in MG cells and tissues and that a nano-DDS can be linked or anchored to MG via EDB-FN-targeting (Figure 6). Although the dose of DTX for orthotopic xenograft animal models was determined as 10 mg/kg in total, which is about 33% less than 15 mg/kg in total for flank xenograft animal models, with consideration of neuronal toxicity of the DTX 39, significant antitumoral efficacy was also presented in the orthotopic models, which was shown in the flank models. Despite the limitations of BBTB, the engineered EDB-FN-targeting micelle nano-DDSs showed significant therapeutic effects compared to the non-targeting micellar nano-DDS, which had no statistically significant antitumoral efficacy. Specific binding of APT_EDB_ to MG cells may have efficiently increased tumor retention time of the APT_EDB_-DSPE micellar nano-DDS, resulting in superior antitumor efficacy. EDB-FN is specifically and highly expressed in MG tissues but is minimally expressed in adjacent normal tissues and normal brain tissues. This characteristic may further contribute to the feasibility of using EDB-FN as a targeting ligand for MG treatment.

## Conclusion

We elucidated the significance of EDB-FN as a potential diagnostic and prognostic biomarker for MG. To the best of our knowledge, no studies comparing EDB-FN expression levels in cancers of all major organs have been conducted, and studies involving prognostic prediction by EDB-FN expression have not been conducted using large bioinformatic datasets. As the results indicate, investigating the EDB-FN expression level in MG patients may afford useful prognostic predictions, and specific targeting of EDB-FN may provide novel therapeutic modalities for the treatment of MG.

## Supplementary Material

Supplementary methods and figures.

## Figures and Tables

**Table 1:** 'Cancer-to-normal' ratio of EDB-FN expression in carcinomas of various organs

Organ	Number of cancer samples	Number of normal samples	'cancer-to-normal' ratio (mean ± std. dev., A.U.)	Cancer vs normal,p value
Brain	2517	723	1.420 ± 0.229	< 0.0001
Head and Neck	360	119	1.561 ± 0.358	< 0.0001
Oral	309	167	1.296 ± 0.323	< 0.0001
Thyroid	338	197	1.374 ± 0.305	< 0.0001
Lung	2502	650	1.195 ± 0.279	< 0.0001
Breast	5516	471	1.284 ± 0.253	< 0.0001
Gastric	934	110	1.168 ± 0.152	< 0.0001
Liver	524	322	1.077 ± 0.206	< 0.0001
Pancreatic	240	98	1.325 ± 0.465	< 0.0001
Colon	2449	500	1.109 ± 0.217	< 0.0001
Adrenal	355	50	0.996 ± 0.171	nonsignificant
Renal	504	195	1.181 ± 0.196	< 0.0001
Ureter	58	45	0.919 ± 1.058	nonsignificant
Ovarian	1146	92	1.265 ± 0.324	< 0.0001
Uterine	387	192	1.103 ± 0.329	< 0.05
Prostate	257	75	1.419 ± 0.528	< 0.0001
Skin	454	488	1.042 ± 0.173	< 0.0001

Statistical analysis: Mann-Whitney test. A.U: arbitrary unit; std. dev: standard deviation.

**Table 2:** EDB-FN expression in normal brain and various brain pathologies

Pathology	Number of samples	Mean	Standard deviation	Difference from normal (fold)	Difference from normal, p value
Normal	723	0.408	0.132	-	-
AD	227	0.329	0.171	0.808	< 0.0001
MDD	134	0.454	0.048	1.114	< 0.001
Epilepsy	43	0.440	0.120	1.079	< 0.05
PD	51	0.429	0.088	1.054	nonsignificant
Grade I	88	0.534	0.124	1.311	< 0.0001
Grade II	261	0.549	0.076	1.347	< 0.0001
Grade III	310	0.573	0.069	1.406	< 0.0001
Grade IV	274	0.627	0.099	1.539	< 0.0001
MGMT methylated	44	0.608	0.038	1.492	< 0.0001
MGMT unmethylated	34	0.610	0.031	1.497	< 0.0001
IDH-1 mutated	136	0.547	0.060	1.341	< 0.0001
IDH-1 wild-type	79	0.569	0.052	1.397	< 0.0001
Astrocytoma, grade I	74	0.535	0.127	1.312	< 0.0001
Astrocytoma, grade II	134	0.552	0.083	1.355	< 0.0001
Astrocytoma, grade III	132	0.586	0.070	1.439	< 0.0001
GBM, grade IV	865	0.625	0.094	1.534	< 0.0001
GBM, MGMT methylated	44	0.608	0.038	1.492	< 0.0001
GBM, MGMT unmethylated	34	0.610	0.031	1.497	< 0.0001

Statistical analysis: Mann-Whitney test, GBM: glioblastoma multiforme, MGMT: O6-methylguanine-deoxyribonucleic acid methyltransferase promoter, IDH-1: isocitrate dehydrogenase-1.

## Abbreviations

AD: Alzheimer's disease
APT_EDB_: EDB-FN-specific aptamer-like peptide (aptide)
APT_EDB_-DSPE: APT_EDB_-conjugated PEG_2000_-DSPE
APT_EDB_-DSPE-DTX: DTX-loaded APT_EDB_-DSPE micellar nano-DDS
BBTB: blood-brain tumor barrier
DAPI: 4',6-diamidino-2-phenylindole
DDS: drug delivery system
DLS: dynamic light scattering
DSPE: 1,2-distearoyl-sn-glycero-3-phosphoethanolamine
DSPE-DTX: DTX-loaded PEG_2000_-DSPE micellar nano-DDS
DTX: docetaxel
EDA-FN: fibronectin with extra-domain A
EDB-FN: extra-domain B of fibronectin
GBM: glioblastoma multiforme
IDH-1: isocitrate dehydrogenase-1
IIICS-FN: type III connecting segment
Mal-PEG_2000_-DSPE: N-maleimide-PEG_2000_-DSPE (ammonium salt)
MDD: major depressive disorder
MG: malignant glioma
MGMT: O^6^-methylguanine-DNA methyltransferase
PD: Parkinson's disease
PEG_2000_: polyethylene glycol (_2000_)
PEG_2000_-DSPE: polyethylene glycol (_2000_)-DSPE (ammonium salt)
Rh-DSPE: 1,2-dioleoyl-*sn*-glycero-3-phosphoethanolamine-N-(lissamine rhodamine B sulfonyl) (ammonium salt)
UPC: universal expression code
