# Supplementary material for: Extra-domain B of fibronectin as an alternative target for drug delivery and a cancer diagnostic and prognostic biomarker for malignant glioma
Source: Theranostics. 2021 Jan 1;11(2):941–57. doi: 10.7150/thno.44948 (PMC7738868; doi:10.7150/thno.44948)
Supplement: Supplementary file 1 — Supplementary methods and figures. [file thnov11p0941s1.pdf]

Supplementary Information

Extra-domain B of fibronectin as an alternative target for drug delivery and a cancer diagnostic and prognostic biomarker for malignant glioma

Phei Er Saw[1†], Xiaoding Xu[1†], Bo Ram Kang[3,4†], Jungsul Lee[5], Yeo Song Lee[3,4], Chungyeul Kim[6], Hyungsin Kim[7], Shin-Hyuk Kang[7], Yoo Jin Na[3,4], Hong Joo Moon[4], Joo Han Kim[4], Youn-Kwan Park[4], Wonki Yoon[4,8], Jong Hyun Kim[4,8], Taek-Hyun Kwon[4,8], Chulhee Choi[5], Sangyong Jon[2], Kyuha Chong[3,4,8,9*]

[1]Guangdong Provincial Key Laboratory of Malignant Tumor Epigenetics and Gene Regulation, Sun Yat-Sen Memorial Hospital, Sun Yat-Sen University, Guangzhou 510120, People's Republic of China

[2]Department of Biological Sciences, KAIST, 291 Daehak-ro, Yuseong-gu, Daejeon, 34141, Republic of Korea

[3]Photo-Theranosis and Bioinformatics for Tumor Laboratory, Korea University Guro Hospital, Korea University Medicine, Korea University College of Medicine, 148 Gurodong-ro, Guro-gu, Seoul, 08308, Republic of Korea

[4]Department of Neurosurgery, Korea University Guro Hospital, Korea University Medicine, Korea University College of Medicine, 148 Gurodong-ro, Guro-gu, Seoul, 08308, Republic of Korea

[5]Department of Bio and Brain Engineering, KAIST, 291 Daehak-ro, Yuseong-gu, Daejeon, 34141, Republic of Korea

[6]Department of Pathology, Korea University Guro Hospital, Korea University Medicine, Korea University College of Medicine, 148 Gurodong-ro, Guro-gu, Seoul, 08308, Republic of Korea

[7]Department of Neurosurgery, Korea University Anam Hospital, Korea University Medicine, Korea University College of Medicine, 73 Goryeodae-ro, Seoungbuk-gu, Seoul, 02841, Republic of Korea

[8]Neurological Institute, Korea University Guro Hospital, Korea University Medicine, 148 Gurodong-ro, Guro-gu, Seoul, 08308, Republic of Korea

[9]Graduate School of Medical Science and Engineering, KAIST, 291 Daehak-ro, Yuseong-gu, Daejeon, 34141, Republic of Korea

[†]These authors contributed equally to this work.

[*]Corresponding author

Kyuha Chong

Email: kyuhaj@korea.ac.kr

Tel: +82-2-2626-1977

Fax: +82-2-2626-1976

Methods

Competition assay

EDB-FN high expression cells (U87MG and U251MG) and EDB-FN low expression cells (MCF7 and B16F1) were grown on glass coverslips until reaching ~80% confluence. Cells were then pre-treated with different concentrations (100 μg/mL and 500 μg/mL) of free APT_{EDB} peptide for 30 min. Then, rhodamine B-labeled APT_{EDB}-DSPE was added and cells were incubated for an additional 30 min. Thereafter, cells were washed with PBS, fixed with 4% (w/v) paraformaldehyde, and mounted on microscope slides for viewing under a confocal microscope.

Transfection of small interfering RNA (siRNA)

Specific siRNAs for EDB-FN and the scrambled control siRNA were purchased from Bioneer (Daejeon, Republic of Korea). Target sequences of EDB-FN siRNAs used in RNA interference were as follows: sense; ACAGUCCCAGAUCAUGGAG, antisense; CUCCAUGAUCUGGGACUGU. For transfection experiments with Lipofectamine 2000 (Invitrogen, CA, USA), cells were seeded into 96-well or 6-well plates at 60–70% confluence after overnight growth. Lipofectamine-siRNA complexes were prepared according to the manufacturer's instructions. Transfection efficiency was analyzed 48 h later.

In vitro ***cellular uptake of the APT_{EDB}-DSPE micellar nano-DDS***

To confirm intracellular uptake dependent on the amount of EDB-FN expression, U87MG cells were transfected with control siRNA or EDB-FN siRNA as described above. They were incubated for 1 h at 37 °C with 100 μg/mL PEG_{2000}-DSPE micellar nano-DDS or APT_{EDB}-DSPE micellar nano-DDSs with an APT_{EDB}-DSPE concentration of 1.0 wt%, stained

through immunocytochemistry using an antibody against EDB-FN, and mounted with DAPI–containing mounting solution. To confirm the time-dependent cellular uptake, U87MG cells were treated with 100 μg/mL APT_{EDB}-DSPE micellar nano-DDSs with an APT_{EDB}-DSPE concentration of 1.0 wt% for 5 min, 15 min, 30 min, 1 h, and 4 h.

In vivo uptake and toxicity of APT_{EDB}-DSPE.

To evaluate the tissue uptake of the APT_{EDB}-DPSE micellar nano-DDS *in vivo*, U87MG cells were injected into the right flank of BALB/c nude mice (n = 3 mice per group) at 5×10^6 cells/mouse. After 3 weeks, tumor growth was measured, and the tumor volumes were determined to be 80–120 mm^3. Then, 200 μg of the PEG_{2000}-DSPE micellar nano-DDS or APT_{EDB}-DSPE micellar nano-DDS was injected into each mouse, and at predetermined time points (6, 12, 24, and 48 h), the tumor uptake of rhodamine B-labeled micelles was compared using an IVIS *in vivo* imaging system (PerkinElmer, MA, USA).

To verify the safety of APT_{EDB}-DSPE, mouse weight was confirmed before and after the experiment. After the end of the experiment, we euthanized the mice to collect all major organs (heart, liver, spleen, lung, and kidney) for H&E staining.

Immunohistochemistry for frozen samples of orthotopic xenograft model

The brain slices of orthotopic xenograft model attached to the slide glass were washed twice with cold PBS, and then blocked and permeabilized in blocking buffer (PBS containing 0.3% Triton X-100 and 2% BSA) for 1 h. The primary antibody specific for EDB-FN (ab154210; Abcam, MA, USA) was diluted 1:100 in blocking buffer and incubated overnight at 4 °C with the tissue. After washing with PBS, it was incubated for 1 hour at room temperature with Alexa Fluor 488 conjugated secondary antibody (A11001; Invitrogen, CA, USA) diluted 1:200 in blocking buffer. The tissue was counterstained with 4',6-

diamidino-2-phenylindole (DAPI; Invitrogen, NY, USA), covered with a cover slide, and analyzed using a confocal laser scanning microscope and slide scanner (Axio Scan.Z1; Carl Zeiss, NY, USA).

Supplementary Table

Table S1. EDB-FN expression-related prognostic differences among GBM patients

Dataset	Number of samples	Z-value	Dataset	Number of samples	Z-value
**PMID-18772890-TCGA**	203	-1.34	**GSE43378**	32	-1.40
**GSE84010**	349	-0.13	**GSE4271**	56	-0.26
**GSE83300**	50	-3.08	**GSE42669**	55	1.57
**GSE83294**	59	2.00	**GSE33331**	21	0.31
**GSE82009**	28	-1.93	**GSE30472**	29	-1.03
**GSE79671**	36	-0.53	**GSE26576**	25	1.05
**GSE7696**	80	1.21	**GSE1993**	39	-0.16
**GSE74187**	60	-1.41	**GSE18166**	81	0.49
**GSE73038**	21	0.02	**GSE13041**	27	-0.31
**GSE72951**	110	0.54	**GSE108474**	210	0.58
**GSE61335**	44	-2.48	**Integrated Z-value (by the Lipták method)**	**- 0.97 (p < 0.33)**	

EDB-FN: extra-domain B of fibronectin, GBM: glioblastoma multiforme.

Supplementary Figures

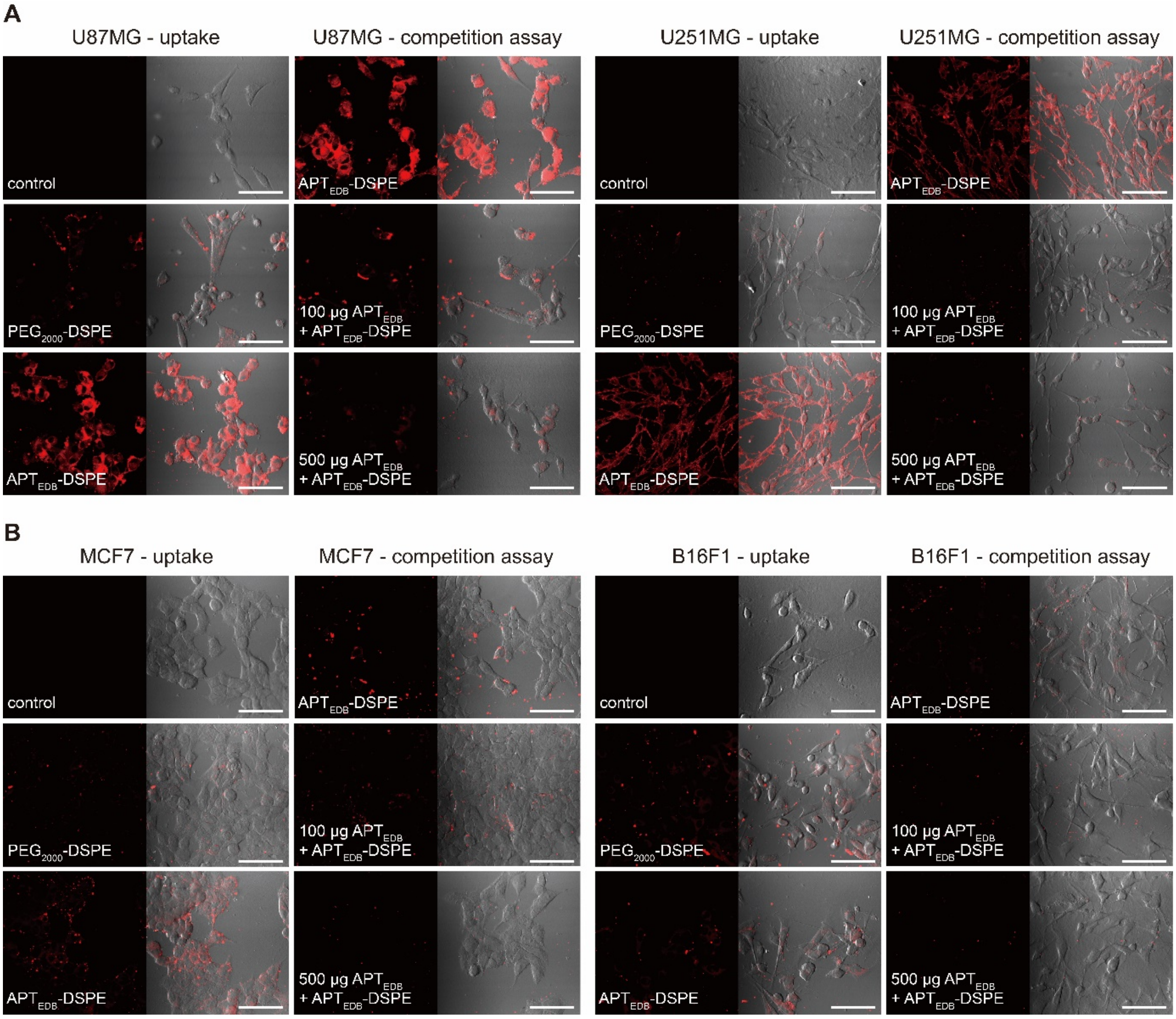

Figure S1. *In vitro* cellular uptake of APT$_{EDB}$-DSPE micellar nano-DDS by EDB-FN expression. Uptake experiments and competition assay of PEG$_{2000}$-DSPE and APT$_{EDB}$-DSPE were performed in **(A)** EDB-FN high expression cells (U87MG and U251MG) and **(B)** low expression cells (MCF7 and B16F1). Phosphate buffered saline for control, PEG$_{2000}$-DSPE, and APT$_{EDB}$-DSPE were treated to the cell lines with or without APT$_{EDB}$, and the uptake of rhodamine B-labeled micellar DDSs (red) was confirmed with a confocal microscope. Scale bar = 100 µm. APT$_{EDB}$: EDB-FN–specific aptamer-like peptide (aptide); APT$_{EDB}$-DSPE: APT$_{EDB}$-conjugated PEG$_{2000}$-DSPE; PEG$_{2000}$-DSPE: polyethylene glycol ($_{2000}$)-1,2-distearoyl-*sn*-glycero-3-phosphoethanolamine.

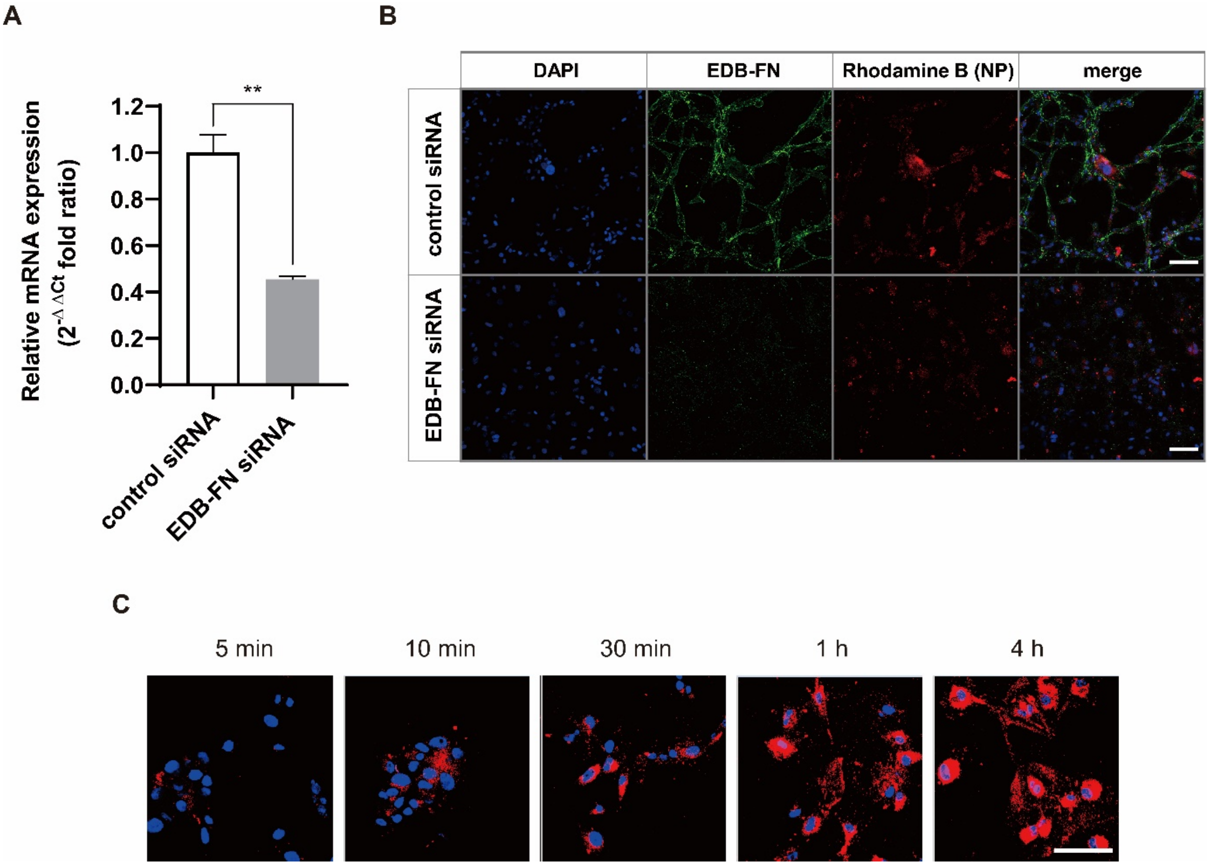

Figure S2. ***In vitro*** **EDB-FN– and time–dependent cellular uptake of APT$_{\mathbf{EDB}}$-DSPE micellar nano-DDS. (A)** The siRNA-mediated EDB-FN knocked down confirmation through qRT-PCR. GAPDH expression was used as internal control. Statistical analysis: Welch's *t* test. $^{**}p < 0.01$. The results are presented as the mean values ± standard deviation of triplicate determinations. **(B)** Cellular uptake of the APT$_{EDB}$-DSPE micellar nano-DDS according to EDB-FN expression. Representative staining images for EDB-FN (green), rhodamine B-labeled APT$_{EDB}$-DSPE micellar nano-DDS (red), and nuclei (blue) in siRNA transfected U87MG cells. Scale bar = 100 μm. **(C)** Time-dependent cellular uptake of the APT$_{EDB}$-DSPE micellar nano-DDS in U87MG cells. Red: rhodamine B-labeled APT$_{EDB}$-DSPE micellar nano-DDS, Blue: DAPI, Scale bar = 100 μm. NP: nanoparticle.

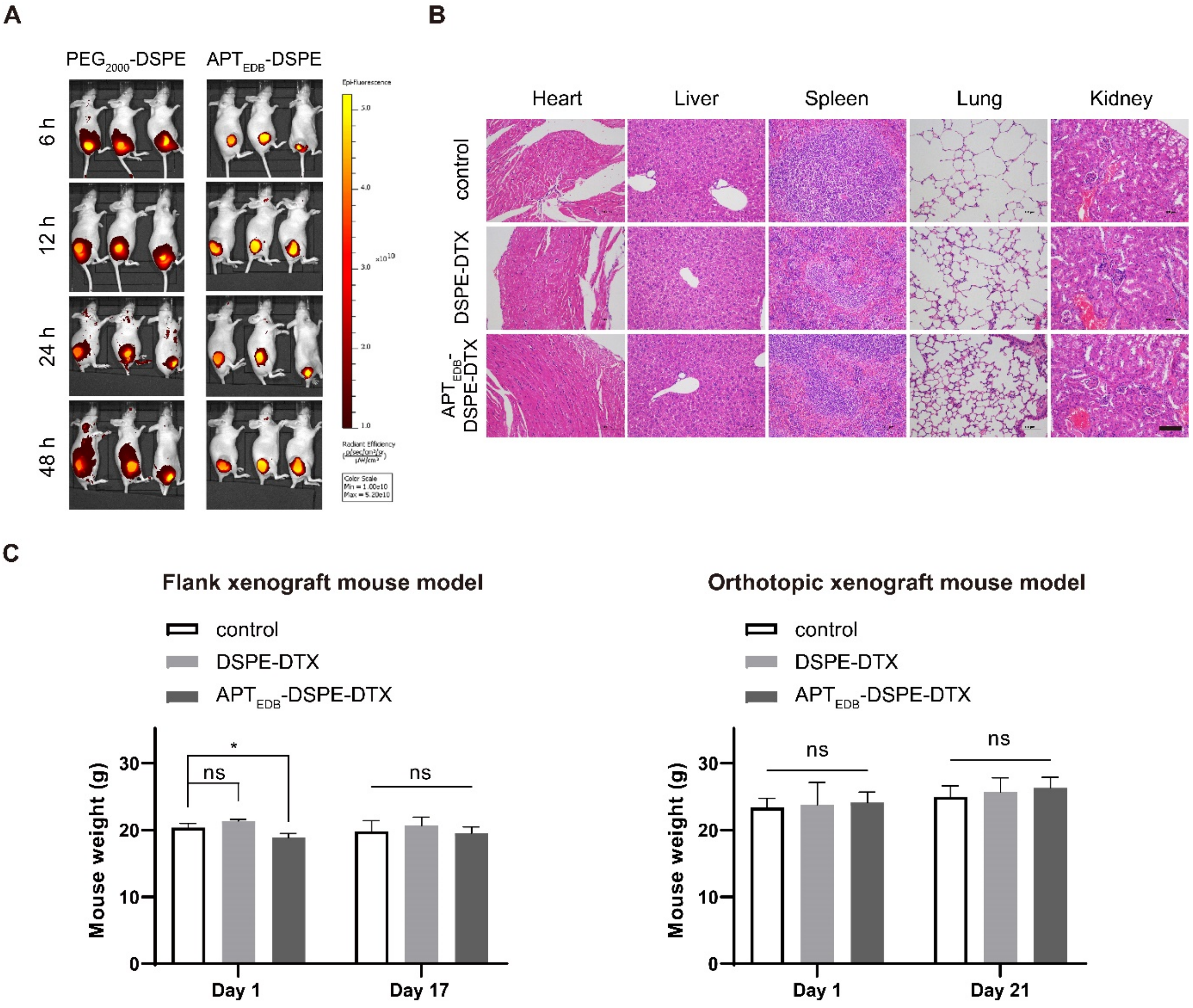

Figure S3. The biocompatibility of APT$_{EDB}$-DSPE-DTX *in vivo.* (A) Cancer targeting ability of APT$_{EDB}$-DSPE-DTX. The PEG$_{2000}$-DSPE micellar nano-DDS or APT$_{EDB}$-DSPE micellar nano-DDS was injected into each U87MG flank xenografted mouse (n = 3 mice per group), and at predetermined time points (6, 12, 24, and 48 h), the tumor uptake of rhodamine B-labeled micelles was compared using an IVIS *in vivo* imaging system. Scale bar = 1 cm. **(B)** Minimal toxicity of micelle nano-DDSs to normal major organs. At the end of the experiment (day 17, once the tumors reached a volume of 80–120 mm^3), the heart, liver, spleen, lung, and kidney were extracted from U87MG flank xenograft mouse models, and H&E staining was performed. Scale bar = 100 μm. **(C)** Minimal toxicity of micelle nano-DDSs to mouse weight in U87MG subcutaneous xenograft model (left; n = 3 mice per group) and in U87MG orthotopic xenograft

model (right; n = 4 mice per group). During the experiment, the mice were weighed every day, and the mice weights at the start and end of the experiment were shown as graphs. Statistical analysis: Welch's *t* test. *$p < 0.05$. The results are presented as the mean values ± standard deviation. APT_{EDB}-DSPE-DTX: docetaxel-loaded APT_{EDB}-DSPE micellar nano-DDS; DSPE-DTX: docetaxel-loaded PEG_{2000}-DSPE micellar nano-DDS.

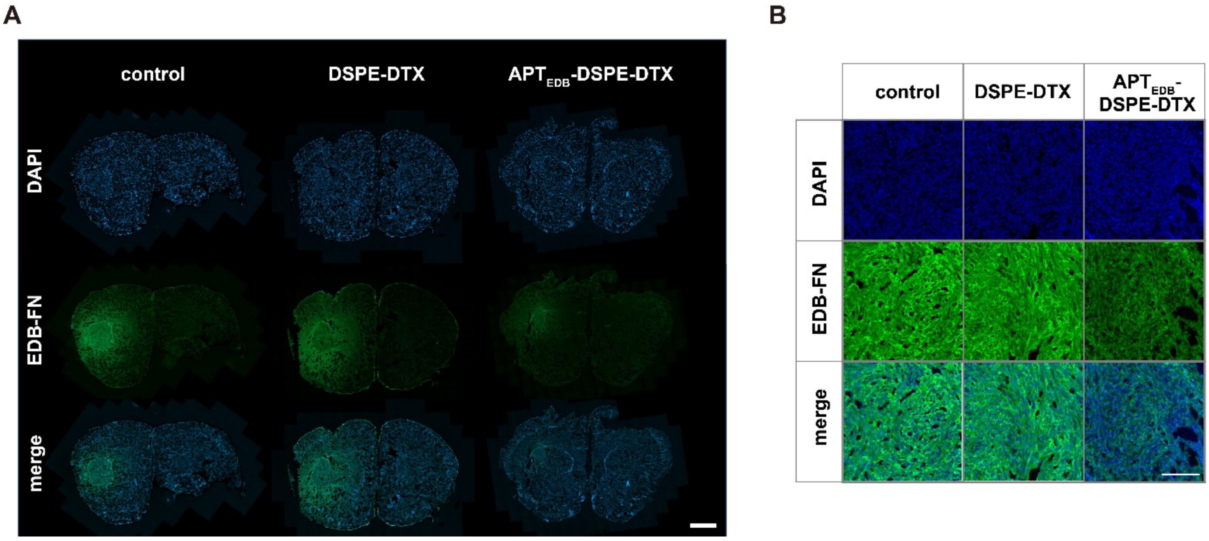

Figure S4. The EDB-FN expression in orthotopic xenograft model. Overexpression of EDB-FN in orthotopic xenografted brain tumor. The model mice injected with saline (control), DSPE-DTX, or APT_{EDB}-DSPE-DTX micellar nano-DDS for 2 weeks. The mouse brain was sectioned to a thickness of 20 μm. **(A)** IHC image was represented by EDB-FN (green) and Nuclei (blue). Scale bar = 1 mm. **(B)** An enlarged image of tumor area. Scale bar = 100 μm. APT_{EDB}-DSPE-DTX: docetaxel-loaded APT_{EDB}-DSPE micellar nano-DDS; DSPE-DTX: docetaxel-loaded PEG_{2000}-DSPE micellar nano-DDS.
